# Identifying a novel mechanism of L-leucine uptake in *Mycobacterium tuberculosis* using a chemical genomic approach

**DOI:** 10.7554/eLife.107025

**Published:** 2026-09-23

**Authors:** Nisheeth Agarwal, Himanshu Gogoi, Eeba, Linus Augustin, Mohd Younus Khan, Yashwant Kumar, Sayan Kumar Bhowmick, Bappaditya Dey

**Affiliations:** 1 Translational Health Science and Technology Institute, NCR Biotech Science Cluster Faridabad India; 2 National Institute of Animal Biotechnology (NIAB) Hyderabad India; Indian Institute of Science India; University of the Witwatersrand South Africa

**Keywords:** *Mycobacterium tuberculosis*, drug-repurposing, semapimod, amino-acid uptake, PDIM, L-leucine uptake, Other

## Abstract

Amino acid biosynthesis is vital for *Mycobacterium tuberculosis* (Mtb) proliferation and tuberculosis (TB) pathogenesis. However, it is not clear how amino acids are transported in Mtb, particularly the branched-chain amino acids (BCAAs) that contribute to the production of the cell-wall lipid component precursors, such as acetyl-CoA and propionyl-CoA. While performing the screening of an FDA-approved repurposed library of small molecule inhibitors against the auxotrophic strain Mtb mc^2^ 6206, which lacks *leuC-leuD* and *panC-panD* genes, we identified a molecule, namely semapimod, which exclusively inhibits the growth of the auxotrophic strain, whereas no effect is observed against the wild-type Mtb H_37_Rv. Interestingly, 24 hr of exposure of Mtb mc^2^ 6206 to semapimod causes massive transcriptional reprogramming with differential expression of >450 genes associated with a myriad of metabolic activities. By performing a series of experiments, we affirm that semapimod indeed inhibits the L-leucine uptake in Mtb mc^2^ 6206 by targeting a protein involved in the cell-wall lipid biosynthesis pathway. Remarkably, semapimod treatment of mice infected with Mtb H_37_Rv causes a significant reduction of bacterial load in lungs and spleen, despite showing no efficacy against the pathogenic strain in vitro. Overall findings of our study reveal that together with an endogenous pathway for L-leucine biosynthesis, a well-orchestrated machinery for its uptake is functional in Mtb, which is important for intracellular survival of the TB pathogen.

## Introduction

Tuberculosis (TB), caused by the pathogen *Mycobacterium tuberculosis* (Mtb), is the leading cause of death due to microbial infections. As per the recent estimates, approximately 25% of the global population is asymptomatically infected with Mtb, and about 11 million people developed the disease annually, resulting in the loss of ~1.25 million lives. In 2023, eight countries accounted for more than two-thirds of the global total burden, with India leading the count (WHO, 2024).

TB is curable, and the usual treatment of pulmonary TB (PTB) cases involves four antibiotics (isoniazid [H], rifampicin [R], pyrazinamide [Z], and ethambutol [E]) for the first 2 months followed by 4 months of treatment with R and H (WHO, 2024; Rendon et al., 2016; Caminero and Scardigli, 2015; Caminero et al., 2010). Unfortunately, due to multiple reasons such as delayed or misdiagnosis, inadequate availability of drugs in resource-limited settings, poor administration, and noncompliance with the prolonged toxic drug regimen, TB becomes resistant to these drugs (Akalu et al., 2024; Bakshi et al., 2011; Hapolo et al., 2019). The drug-resistant TB (DR-TB) is classified under five categories: H-resistant TB (Jhun and Koh, 2020); R-resistant TB (RR-TB) (Malenfant and Brewer, 2021); multidrug-resistant TB (MDR-TB), which is resistant to both H and R; pre-extensively drug-resistant TB (pre-XDR-TB), which is resistant to H, R, and any fluoroquinolone or any of the second-line injectables, such as amikacin, capreomycin, and kanamycin; and XDR-TB, which is resistant to H, R, any fluoroquinolone, and a second-line injectable or at least one of the recently developed drugs, bedaquiline and linezolid. Treatment of DR-TB involves lengthy treatment with a course of second-line drugs for 6–18 months, causing huge socioeconomic burden (Mase and Chorba, 2019). DR-TB continues to be a threat to the public health system as the number of people developing MDR/RR-TB remains stable since 2020. Globally, ~400,000 MDR/RR-TB cases were reported in 2023, with about one-fourth of the global MDR-TB cases reported from India alone (WHO, 2024; Muniyandi and Ramachandran, 2017). Considering the grim situation of everlasting DR-TB cases and emergence of resistance to newly developed drugs, there is a pressing need to explore novel therapeutic approaches.

With advancements in medicinal chemistry, large numbers of new chemical entities (NCEs) have been synthesized over the last three decades that were evaluated for antimicrobial activity by the target-based and the phenotypic screening approaches (Boyd et al., 2021; Kim et al., 2025). However, only a few of the newly developed antibiotics that got approval for human use exhibit a novel mechanism of action (MoA), whereas the majority are the derivatives of the existing antibiotic classes (Boyd et al., 2021; Butler et al., 2023). Evaluation of the antibiotic potential of the existing drugs that are approved for human use by the Federal Drug Agency (FDA) for other diseases, also known as drug repurposing, has emerged as an alternative approach to expedite the treatment of infectious diseases, including TB, as it avoids much of the hurdles at the early phases of drug development (Boyd et al., 2021; Beachy et al., 2014).

Herein, we report that a new anti-inflammatory molecule, semapimod, identified from a screen of the repurposed library of FDA-approved drugs, kills Mtb mc^2^ 6206 (a leucine and pantothenate auxotroph) at a reasonably low concentration of 20 nM. To our great surprise, semapimod fails to inhibit other mycobacterial species, including pathogenic Mtb H_37_Rv. These unexpected findings led us to identify a functional L-leucine uptake system in Mtb, which is blocked by the newly identified compound. We show that semapimod targets an enzyme, PpsB, involved in the synthesis of the cell-wall phthiocerol dimycocerosate (PDIM), and influences Mtb virulence during host infection.

## Results

### Screening of the FDA-approved library of molecules against Mtb mc^2^ 6206

**Figure 1.**
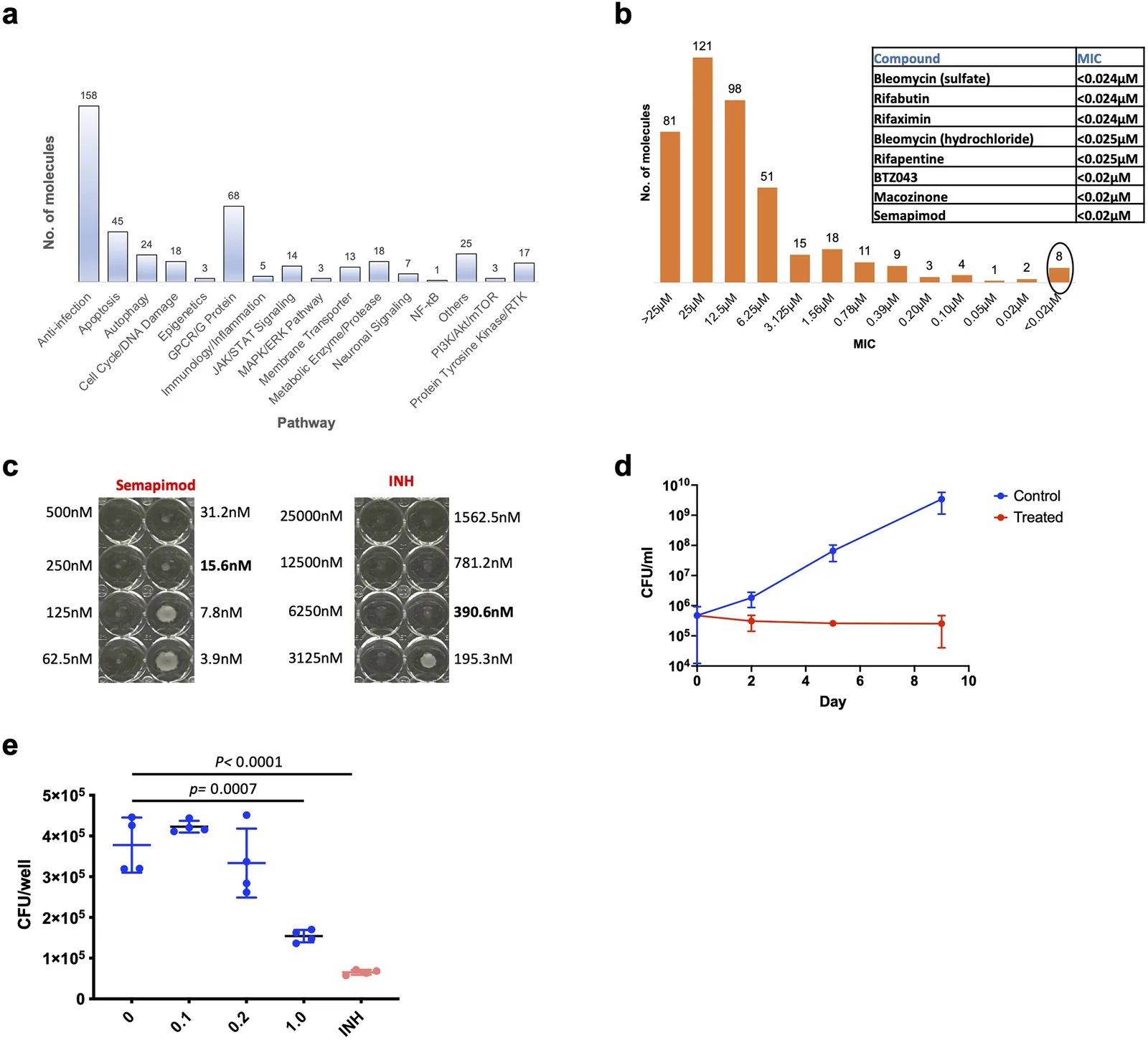
Screening of the FDA-approved library of molecules against Mtb mc^2^ 6206 reveals the growth-inhibitory effect of semapimod, an anti-inflammatory small molecule. (**a**) Status of inhibitors showing activity against Mtb mc^2^ 6206. The bar graph depicts Mtb mc^2^ 6206 inhibitors acting on different host metabolic pathways. (**b**) MIC_90_ status of Mtb inhibitors. The bar graph shows inhibitors with different MIC_90_ against Mtb mc^2^ 6206, in vitro. Molecules with <0.025 µM MIC_90_ (encircled) are tabulated in the inset. (**c**) Determination of the minimum dose of semapimod for complete inhibition of Mtb mc^2^ 6206 growth by visual inspection. Isoniazid (INH) was used as a control in the 96-well plate-based assay. The drug concentration beyond which no visual growth is observed is shown in bold. (**d**) Effect of semapimod on the in vitro growth of Mtb mc^2^ 6206. Shown is the CFU enumeration of drug-treated and untreated (control) bacteria at the indicated time points. (**e**) Effect of semapimod on the intracellular proliferation of Mtb mc^2^ 6206. Intracellular growth of Mtb mc^2^ 6206 in the THP1-derived macrophages was examined after 6 days of infection in the absence (UT) or presence of 0.10–1.0 µM semapimod. Treatment with 2.5 µM INH was used as control. A significant reduction in CFU counts of Mtb mc^2^ 6206 is observed in the presence of semapimod under in vitro culture conditions (**d**) as well as during intracellular (*P*<0.005) growth (**e**). Data represent mean ± SD of at least *n*=2 replicates in **d** and *n*=4 replicates in (**e**). Data in (**c**) are representative of *n*=2 independent experiments. p Values in (**e**) were obtained after comparison of CFUs between the UT and semapimod-treated samples, as described in Materials and methods.

In this study, we screened a repurposed library (MedChemExpress) consisting of 3614 FDA-approved small molecule inhibitors against Mtb mc^2^ 6206, which is commonly used as a surrogate strain for pathogenic Mtb H_37_Rv (Jain et al., 2014). These molecules primarily target microbial infections (790), GPCR/G Protein (646), apoptosis (371), metabolic enzyme (including protease; 355), membrane transporter (236), autophagy (210), cell cycle/DNA damage (157), immunology/inflammation (109), protein tyrosine kinase/RTK (99), and a large number of molecules targeting other miscellaneous pathways (402; Figure 1—figure supplement 1a). The library contains a versatile set of molecules targeting different diseases such as cancer, cardiovascular disease, endocrinology, infection, inflammation/immunology, metabolic disease, and neurological disease (Figure 1—figure supplement 1b). While these are intended for human use, 66% of the molecules have recently been launched, whereas the remaining inhibitors are at different phases of trial, as depicted in Figure 1—figure supplement 1c. Of these, we identified 422 molecules showing complete inhibition of growth at 50 µM concentration. As anticipated, the majority (158) of the Mtb inhibitors show anti-infection activity. However, several other compounds acting on host metabolic pathways, such as GPCR/G protein (68), apoptosis (45), autophagy (24), cell cycle/DNA damage (18), metabolic enzyme/protease (18), protein tyrosine kinase/RTK (17), JAK/STAT signaling (14), and membrane transporter (13) substantially inhibit in vitro growth of Mtb mc^2^ 6206 (Figure 1a).

**Figure 1—figure supplement 1.**
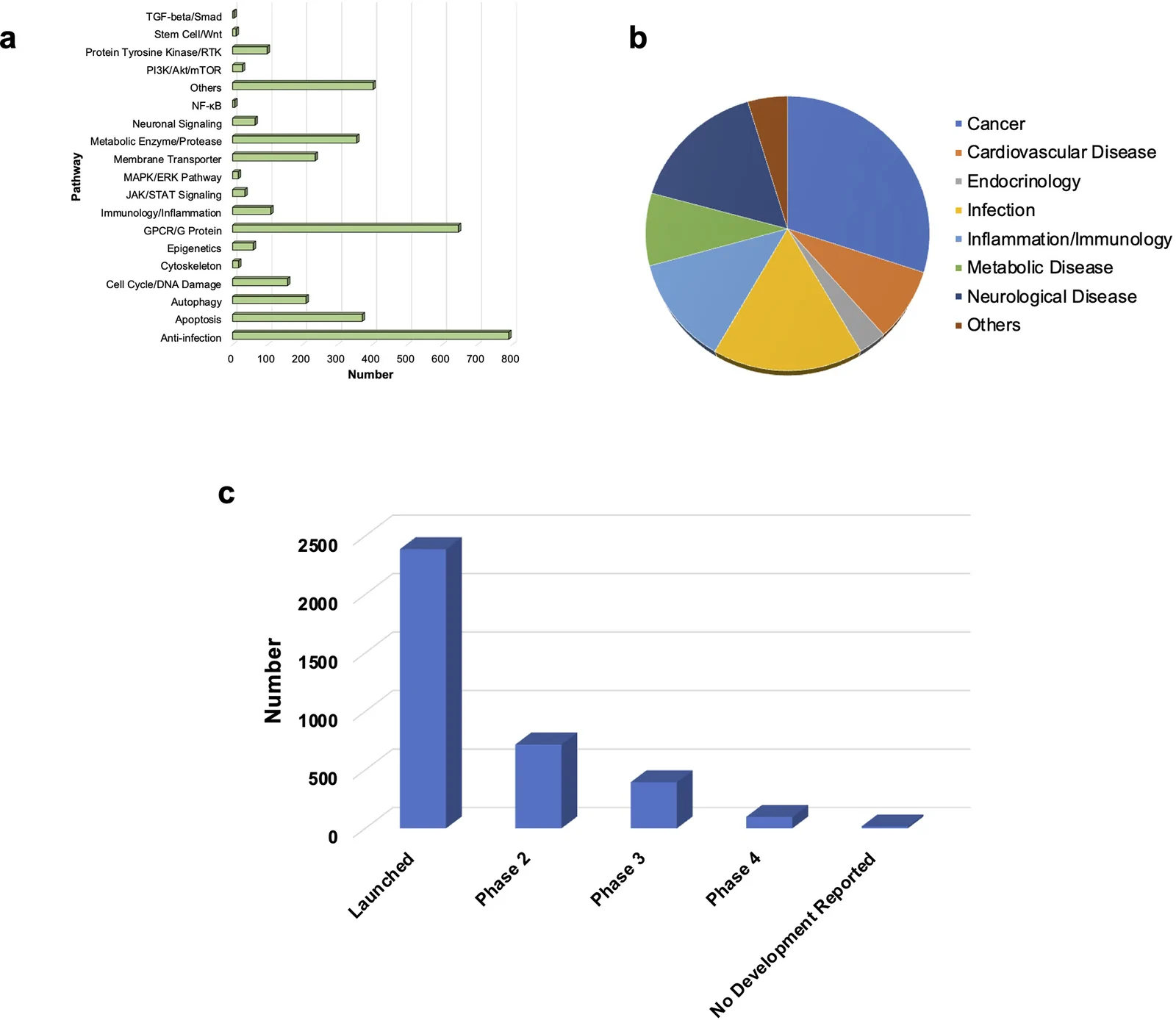
Characteristics of the small molecules’ library. (**a**) The bar graph shows the status of molecules in the library acting on different metabolic pathways. (**b**) The pie chart depicts various diseases that are targeted by inhibitors. (**c**) Shown is the ranking of FDA-approved molecules as per the different clinical trial phases.

Next, we determined the minimum concentration of newly identified anti-mycobacterial compounds at which ~90% growth is suppressed (MIC_90_), by using a plate-based resazurin assay. Our results reveal 300 molecules showing an MIC_90_ of >12.5 µM, whereas the remaining 122 molecules exhibit MIC_90_ values ranging from 6.25 to <0.025 µM (Figure 1b). Surprisingly, out of the eight molecules which inhibit growth at <0.025 µM, we find an anti-inflammatory molecule namely semapimod which demonstrates anti-microbial activity against Mtb mc^2^ 6206. Notably, semapimod is an inhibitor of proinflammatory cytokine production, which suppresses the production of TNF-α, IL-1β, and IL-6 (Nishimatsu et al., 2008; Wehner et al., 2009; Wang et al., 2016; Miller et al., 2014), and its anti-mycobacterial activity has never been reported to the best of our knowledge.

### Semapimod shows bactericidal activity against Mtb mc^2^ 6206 under extra- and intracellular growth conditions

To corroborate the findings from the initial screen, we tested the effect of the fresh batch of semapimod against Mtb mc^2^ 6206, which reveals complete inhibition of mycobacterial growth at ~15 nM (Figure 1c). The colony-forming unit (CFU) estimation further shows a bactericidal activity of this molecule which causes 88% reduction of bacterial viability on day 2 and >99% reduction after 5 days of incubation (Figure 1d). In addition to its effect on in vitro growth, we also find a substantial reduction in viable bacterial counts during macrophage infection. Incubation of Mtb mc^2^ 6206-infected THP1 macrophages with different doses of semapimod reveals 60% reduction (*P*<0.005) in intracellular CFU counts after 6 days of incubation with 1 µM inhibitor (Figure 1e). Notably, no cytotoxic effect was observed at this concentration against THP1, thus ruling out the possibility of cell lysis by semapimod.

### Global transcriptional profile of Mtb in response to semapimod treatment

**Figure 2.**
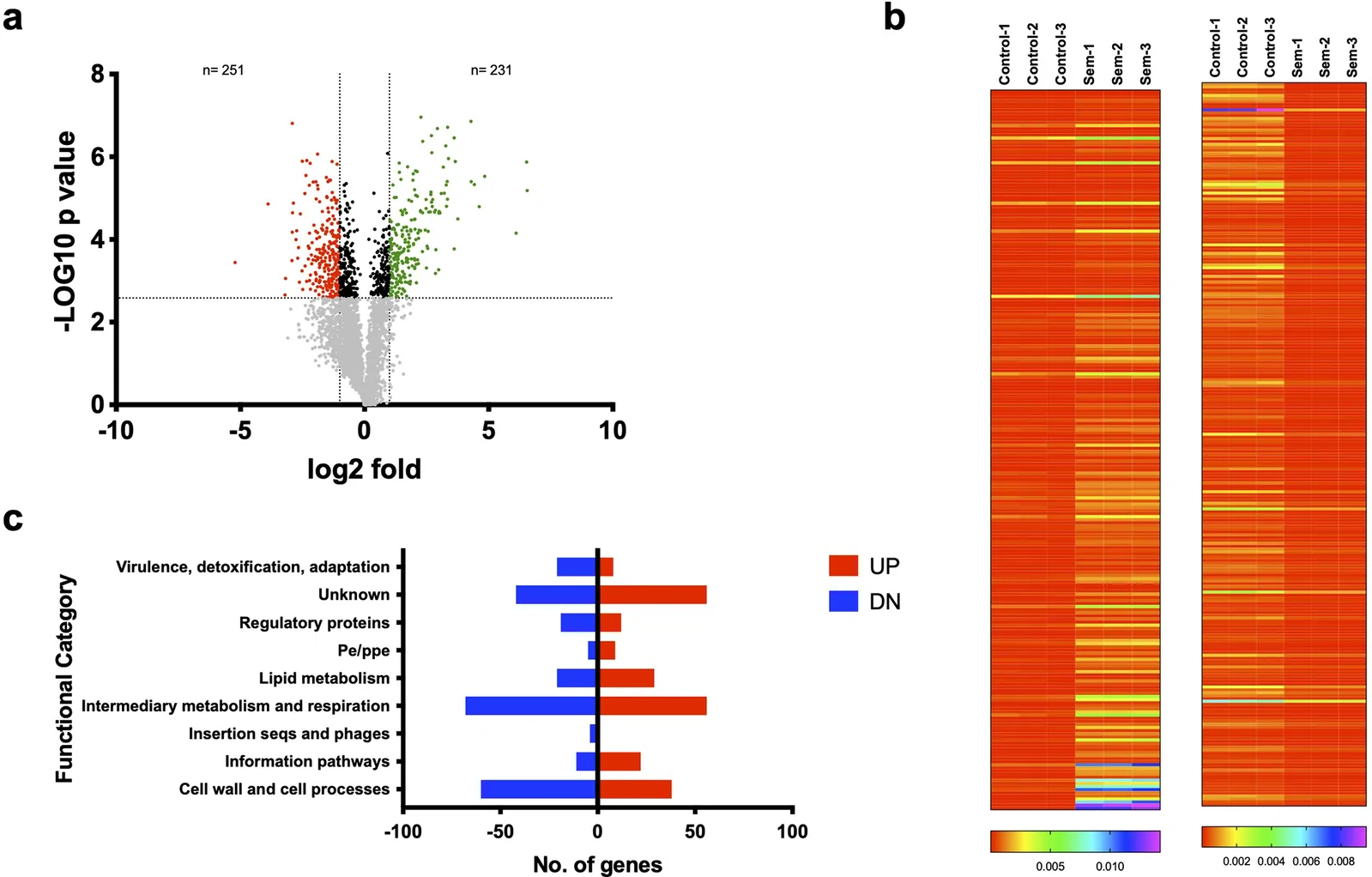
Effect of semapimod treatment on the expression profile of Mtb mc^2^ 6206 transcripts. (**a**) Volcano plot of differentially expressed genes in semapimod-treated Mtb mc^2^ 6206. The plot shows the distribution of genes that are differentially expressed *via* log2 fold-change and the –log *p* values. Broken vertical lines represent the cutoff of >1.0 log2 fold-change, and the horizontal line represents the cutoff of >2.583 –log p values. Genes below the -log p cutoff are represented by grey dots. Downregulated genes are represented by red dots, and those showing upregulation in response to semapimod treatment are shown with green dots. (**b**) Status of differentially accumulated transcripts. Heatmap representation of transcripts showing accumulation (left) or suppression (right) upon exposure to semapimod across three biological replicates. (**c**) Functional categorization of differentially regulated genes. The butterfly chart shows the distribution pattern of differentially regulated genes according to their function, as classified in the Mycobrowser database (https://mycobrowser.epfl.ch/genes/). Mean fold-change values from n=3 biological replicates are shown in (**a**). Figure 2—source data 1.List of differentially expressed genes in semapimod-treated Mtb mc2 6206.Shown are genes exhibiting >2.0-fold change in expression. p Values were determined by using the two-stage setup method of Benjamini, Krieger, and Yekutieli, keeping FDR (**Q**) of 1% with the help of GraphPad Prism v7.0e software.

To gain mechanistic insights into the effect of semapimod on Mtb mc^2^ 6206, we analyzed the global transcriptional profile of the bacterium in response to this drug. Bacterial cultures in the logarithmic growth phase (OD600 of ~0.4) were treated with 50 nM semapimod, equivalent to ~3x MIC, and total RNAs were harvested from semapimod-treated and untreated bacteria after 24 hr of incubation, as described in the Materials and methods. RNA samples from three biological replicates were subsequently used in RNA sequencing (RNASeq), which reveals differential expression of 482 genes by >2.0-fold (false discovery rate [FDR]-adjusted p value of <0.01). While 251 genes are downregulated by semapimod treatment, expression of 231 genes is induced (Figure 2a, Figure 2—source data 1). Remarkably, the effect of semapimod was consistent across three biological replicates (Figure 2b). Genes, prominently affected by semapimod primarily belong to intermediary metabolism and respiration (n=124), cell-wall and cell processes (n=98), lipid metabolism (n=50), and those encoding for regulatory proteins (n=31; Figure 2c). Expression of some of the representative genes was also validated by qRT-PCR using gene-specific primers, which shows a similar pattern of expression (Figure 2—figure supplement 1), thus confirming the specificity of the RNASeq data.

**Figure 2—figure supplement 1.**
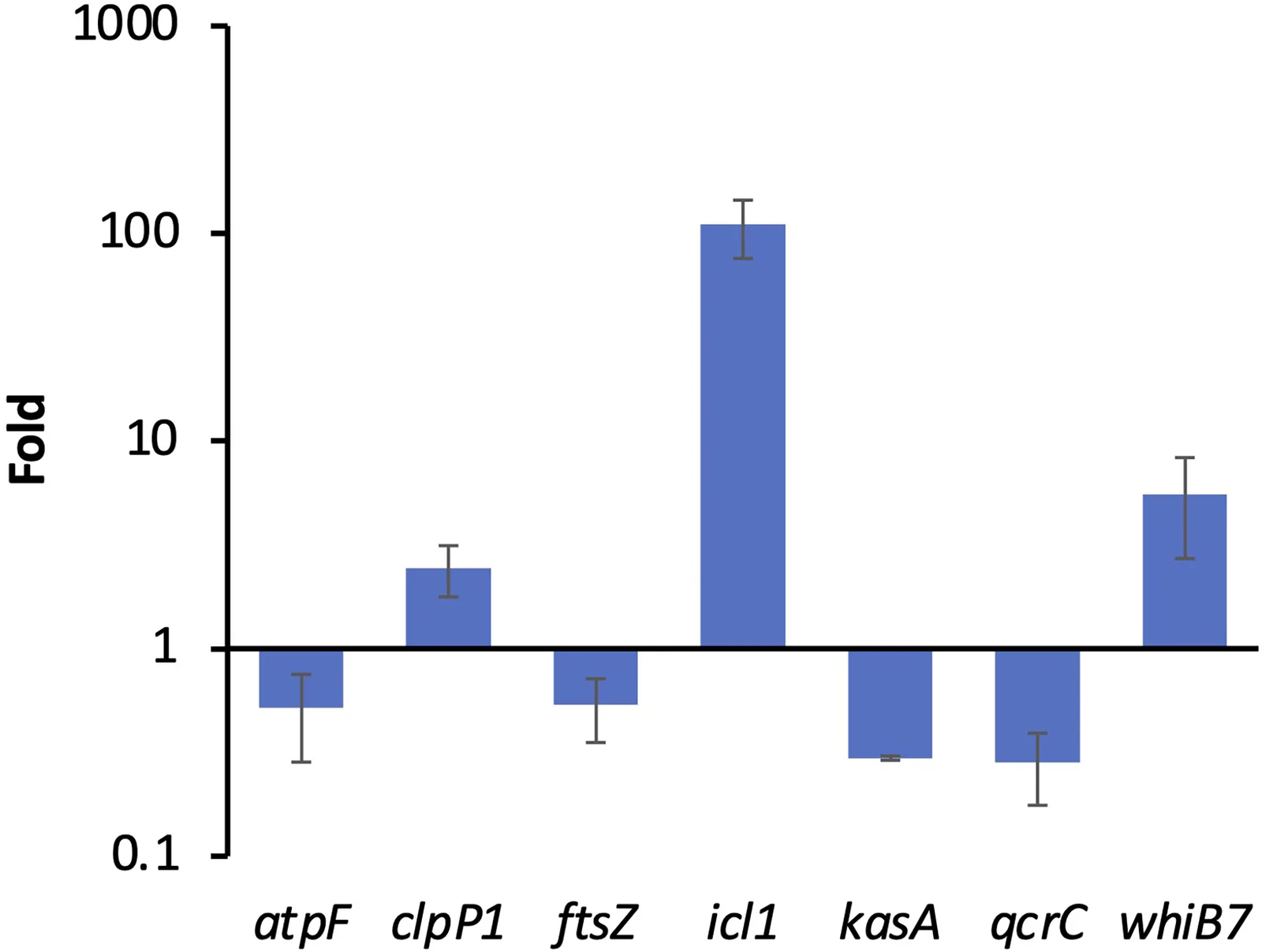
Validation of RNASeq data by qRT-PCR. The bar graph shows the expression levels of representative genes in semapimod-treated bacteria, estimated by qRT-PCR. Fold-change was calculated with respect to untreated samples after normalization with *rrs*, which remained consistent across the two groups. Mean ± SD values of *n*=2 biological replicates are shown.

**Figure 2—figure supplement 2.**
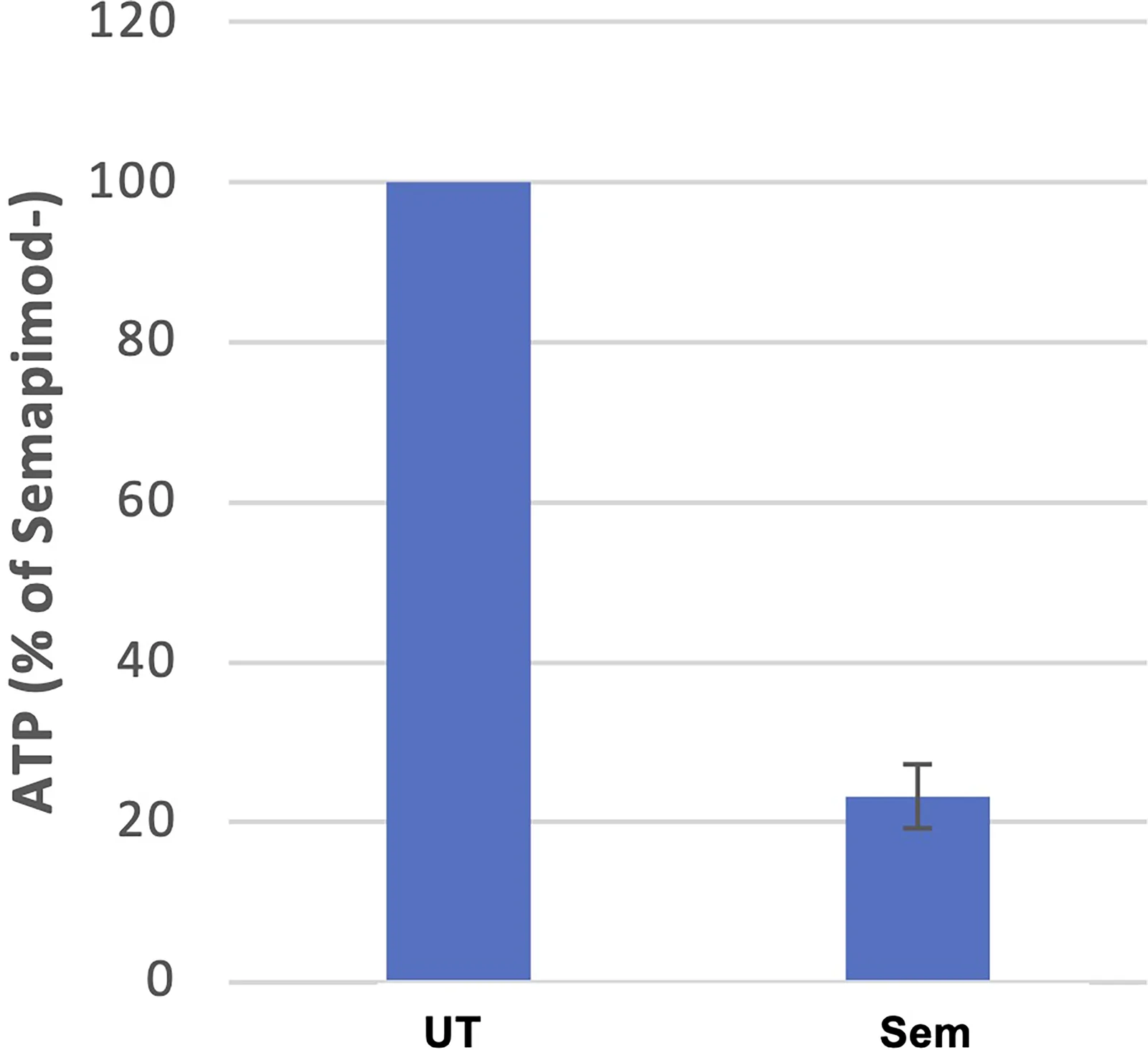
Semapimod treatment causes a reduction in intracellular ATP level. Intracellular ATP was estimated in UT and treated Mtb mc^2^ 6206 exposed to 50 nM semapimod (Sem), using the Bac TiterGlo assay kit, as suggested by the manufacturer (Promega). The bar graph shows the percentage change in the intracellular ATP levels following drug exposure. Mean ± SD values of *n*=2 biological replicates are shown.

Our results show that genes involved in oxidative phosphorylation, such as *atpF*, *atpH*, *atpG*, *atpD*, *cydB*, *cydA*, *Rv1812c,* and *qcrC,* as well as several *nu*o operon genes (*nuoB*, *nuoC*, *nuoD*, *nuoE*, *nuoF*, *nuoG*, *nuoI*, *nuoJ*, *nuoL*, *nuoM,* and *nuoN*), are downregulated in the drug-treated bacteria. Not only are the expression levels of these respiratory genes perturbed, but a significant reduction in cellular ATP levels is observed upon semapimod treatment of Mtb mc^2^ 6206 (Figure 2—figure supplement 2). Furthermore, we find several genes that encode for subunits of RNA polymerase (*rpoA* and *rpoB*) and ribosome (*rplJ*, *rplL*, *rplC*, *rplD*, *rplW*, *rplB*, *rpmC*, *rpsN1*, *rpsH*, *rplF*, *rplR*, *rpmD*, *rplT,* and *rpsD*) exhibiting distinct upregulation in response to the drug treatment, which indicates perturbation of transcription and translation machineries in the drug-treated bacteria. Notably, semapimod treatment also interferes with the expression of a number of genes associated with the metabolism of amino acids, such as branched-chain amino acids (*ilvC*, *ilvB1*, *ilvN*, *leuA*, *bkdB*, *bkdA*, *Rv2499c*, *accA1,* and *accD1*), cysteine (*csd*, *cysM*, *cysO*, *cysE*, *cysK1,* and *sahH*), lysine (*pcd*, *lat*, *lysA*), methionine (*metZ*, *metK,* and *metH*), threonine (*thrB*, *thrC,* and *thrA*), glutamate (*gdh*), glutamine (*glnA1*), and tryptophan (*trpA*).

Among the cell-wall and cell processes functional category, we find a substantial reduction in the expression of genes involved in peptidoglycan biosynthesis (*pbpA*, *ponA1*, *murI*, *dacB1*), cell-wall arabinogalactan linker formation (*wbbL1*), cell-wall arabinan biosynthesis (*embB* and *aftB*), cell-wall LPS biosynthesis (*Rv0225*, *gca*, *gmhA*, *gmhB,* and *hddA*), cell division (*Rv1708*, *scpA*, *ftsZ,* and *gid*), and transport across the bacterial cell membrane (*ctpB*, *glnH*, *ctpE*, *pstA1*, *pstA2*, *narK2*, *Rv1747*, *ansP1*, *fecB,* and *Rv3200c*).

Another category of genes exhibiting notable changes in expression are those involved in the biosynthesis of lipids, such as cell-wall mycolic acid (*accD6*, *fabD*, *kasA*, *kasB*, *fabG4*, *accD4*, *umaA*, and *cmaA2*), methyl-branched (MB) lipids (*ppsB*, *ppsC*, *ppsD*, *mas*, *fadD26*, *fadD29*, *tesB1*, *Rv2953*, *papA1*, *pks2*, *mmpL8*, *fadD23*, *pks3*, *pks4*, *papA3,* and *fadD21*), phospholipids (*cdh*), and triglycerides (*desA3*). In addition, several genes controlling lipid degradation (*fadE5*, *fadE35*, *fadD12*, *fadE19*, *tesB2*, *fadE21*, *fadE24*, *fadB2*, *fadD36*, *fadE15,* and *fadD9*) and transport (*lprK*, *mce1F*, *mce4F*, *mce4D*, *lprN*, and *lucA*) are also modulated in the drug-treated bacteria. Noteworthy to mention, a marked reduction in expression is observed for genes involved in the synthesis of succinyl-CoA via the vitamin B12-dependent methylmalonyl pathway (MMP; *accA3*, *mutA*, *mutB*, *cobB,* and *cobO*), whereas those associated with the methylcitrate cycle (MCC; *prpD*, *prpC*, *icl1,* and *pks11*) exhibit significant upregulation, together indicating perturbation of acetyl-CoA levels in semapimod-treated Mtb mc^2^ 6206.

Furthermore, we find that semapimod treatment modulates the expression of genes encoding a variety of regulatory proteins, such as kinases (*pknA*, *pknG,* and *pknD*), WhiB-family transcription regulators (*whiB2*, *whiB3,* and *whiB7*), hypoxia regulators (*Rv0081*, *dosS,* and *dosR*), regulators of lipid transport (*Rv0302* and *mce1R*), a regulator of MCC genes (*regX3*), and a leucine-responsive transcription regulator (LrpA).

### Semapimod treatment interferes with L-leucine uptake in Mtb

**Figure 3.**
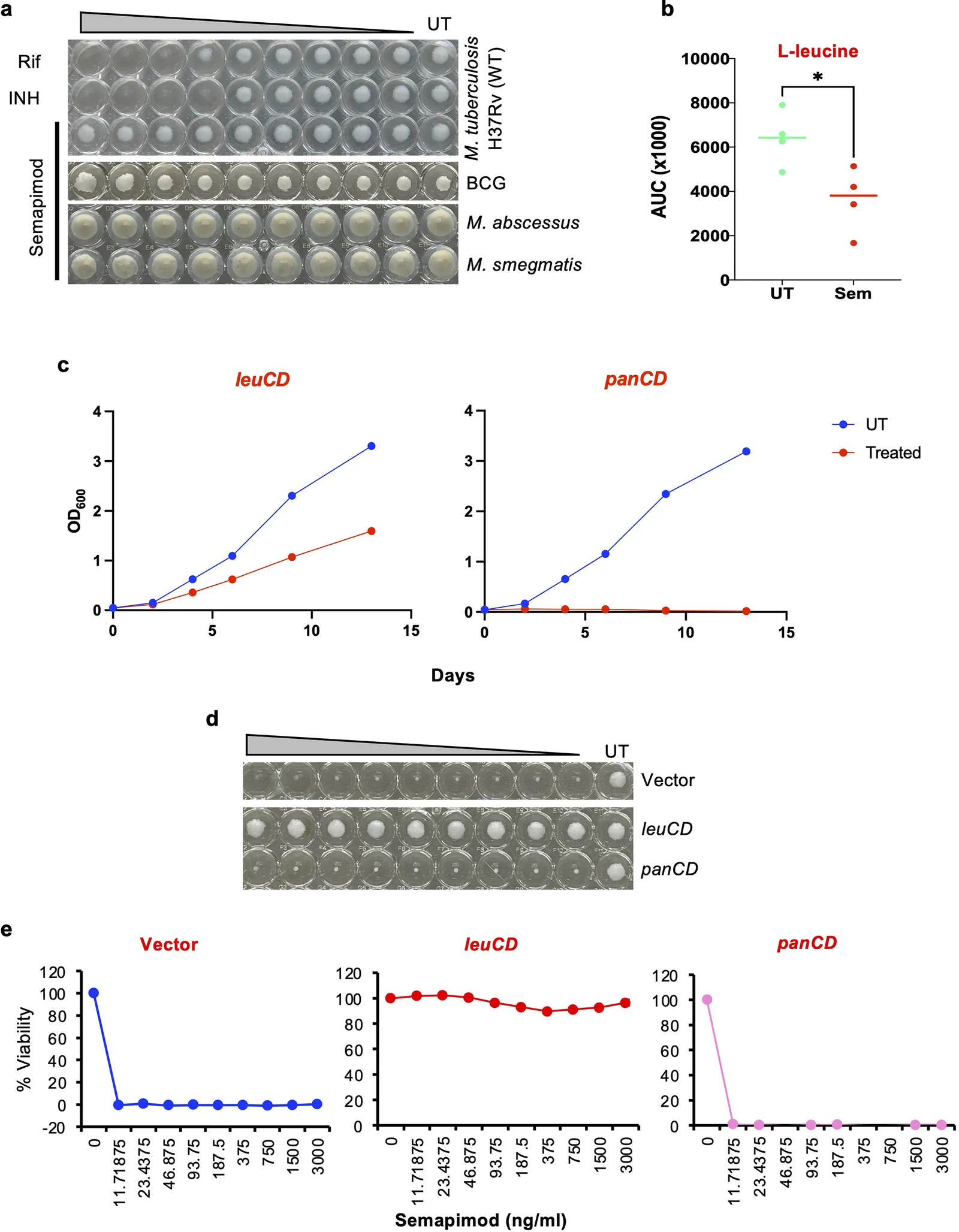
Semapimod treatment perturbs L-leucine uptake in Mtb mc2 6206. (**a**) Effect of semapimod on the in vitro growth of different mycobacterial species. The growth inhibitory effect of semapimod was assessed against slow-growing Mtb H_37_Rv and *M. bovis* BCG, and fast-growing *M. abscessus* and *M. smegmatis*, respectively, by visual inspection using a 96-well plate-based assay. Rifampicin (Rif) and INH were used as control drugs against Mtb H_37_Rv. (**b**) Estimation of intracellular leucine in Mtb mc^2^ 6206. Intracellular leucine was estimated in untreated (UT) and semapimod-treated (Sem) bacteria after 24 hr of drug treatment by mass spectrometry as described in Materials and methods. (**c–e**) Effect of *leuC-leuD* and *panC-panD* expression in Mtb mc^2^ 6206 on bacterial killing by semapimod. Time- (**c**) and dose- (**d–e**) dependent growth kinetics reveal loss of bactericidal effect of semapimod against Mtb mc^2^ 6206 by *leuC-leuD*, and not by *panC-panD* expression. Percent viability in **e** was calculated with respect to untreated (UT) cultures after 2 weeks of exposure to different drug concentrations, using a 96-well plate-based method as described in Materials and methods. Data in (**a**) and (**c–d**) are representative of *n*=2 independent experiments. Mean ± SD values from *n*=4 replicates are shown in (**b**).

To validate the effect of semapimod on the virulent Mtb, we sought to determine its MIC against Mtb H_37_Rv. Surprisingly, the drug was found ineffective against the virulent Mtb strain. Similar to the virulent Mtb strain, no effect was observed against other mycobacterial species such as *Mycobacterium bovis* BCG, *Mycobacterium abscessus,* and *Mycobacterium smegmatis* (Figure 3a). These results pointed out the effect of semapimod on the uptake of leucine and/or pantothenate that are indispensable for the growth of the auxotrophic strain. Altered expression of a set of LrpA-regulated genes (Cho et al., 2008; Duan et al., 2016; Reddy et al., 2008) by semapimod treatment indicates perturbation of leucine uptake. To test this hypothesis, we cultured Mtb mc^2^ 6206 in the absence of pantothenate and L-leucine (PL-), pantothenate (P-), or L-leucine (L-) for 24 hr followed by analysis of the expression of some of the leucine-responsive genes such as *lat*, *leuA*, *lrpA,* and *icl1* as surrogate markers. Interestingly, all these genes exhibit marked increase in expression under PL- and L-, but not under P- conditions (Figure 3—figure supplement 1). A similar expression profile of these genes is observed in semapimod-treated bacteria by RNASeq, thus suggesting the interference of L-leucine uptake. To corroborate these findings, the intracellular leucine concentrations were estimated in the untreated and semapimod-treated Mtb mc^2^ 6206 by mass-spectrometry. Our results show ~45% reduction (p<0.05) in the leucine levels in bacteria exposed to semapimod (Figure 3b). Noteworthy to mention, levels of another BCAA, valine, or an unrelated amino acid, L-proline remain unchanged in Mtb mc^2^ 6206 upon semapimod treatment, which corroborates the effect of the drug specifically on L-leucine uptake (Figure 3—figure supplement 2). Next, we examined the effect of semapimod against Mtb mc^2^ 6206, expressing *leuC-leuD* and *panC-panD*, respectively, under a constitutive promoter. Remarkably, expression of *leuC-leuD*, and not of *panC-panD* genes, alleviates the growth-inhibitory effect of semapimod (Figure 3c–e). Mtb mc^2^ 6206::*leuCD* exhibits comparable growth in the presence and the absence of semapimod (Figure 3c). In addition, Mtb mc^2^ 6206::*leuCD* exhibits resistance to killing by as high as 3.37 µM (>200-fold excess of MIC) semapimod (Figure 3d–e). In contrast, Mtb mc^2^ 6206::*panCD* exhibits a similar level of inhibition by semapimod, as observed with the Mtb mc^2^ 6206. Overall, these results substantiate that semapimod inhibits L-leucine uptake in Mtb.

**Figure 3—figure supplement 1.**
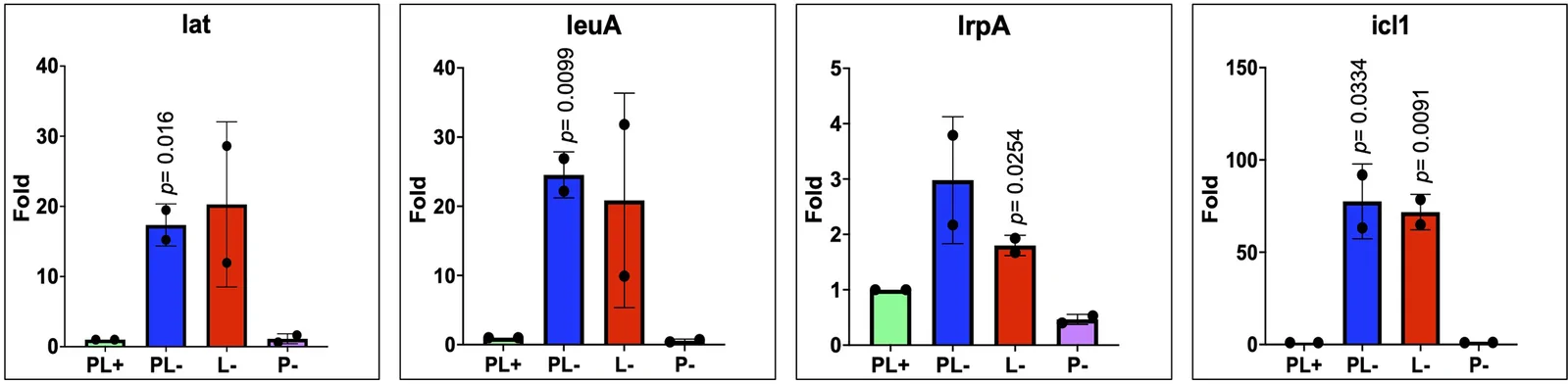
Effect of withdrawal of L-leucine on expression of semapimod-regulated genes in Mtb mc^2^ 6206. The bar graph shows the expression level of various genes in the presence or absence of metabolites, as estimated by qRT-PCR. PL+, 7H9-PLO; PL-, 7H9-O; P-, 7H9-LO; L-, 7H9-PO. Fold-change in expression was calculated in all the samples with respect to PL + after normalization with *sigA*, which remained consistent in all the samples. Mean ± SD values of *n*=2 biological replicates are shown. p Values were calculated using GraphPad Prism version 10.

**Figure 3—figure supplement 2.**
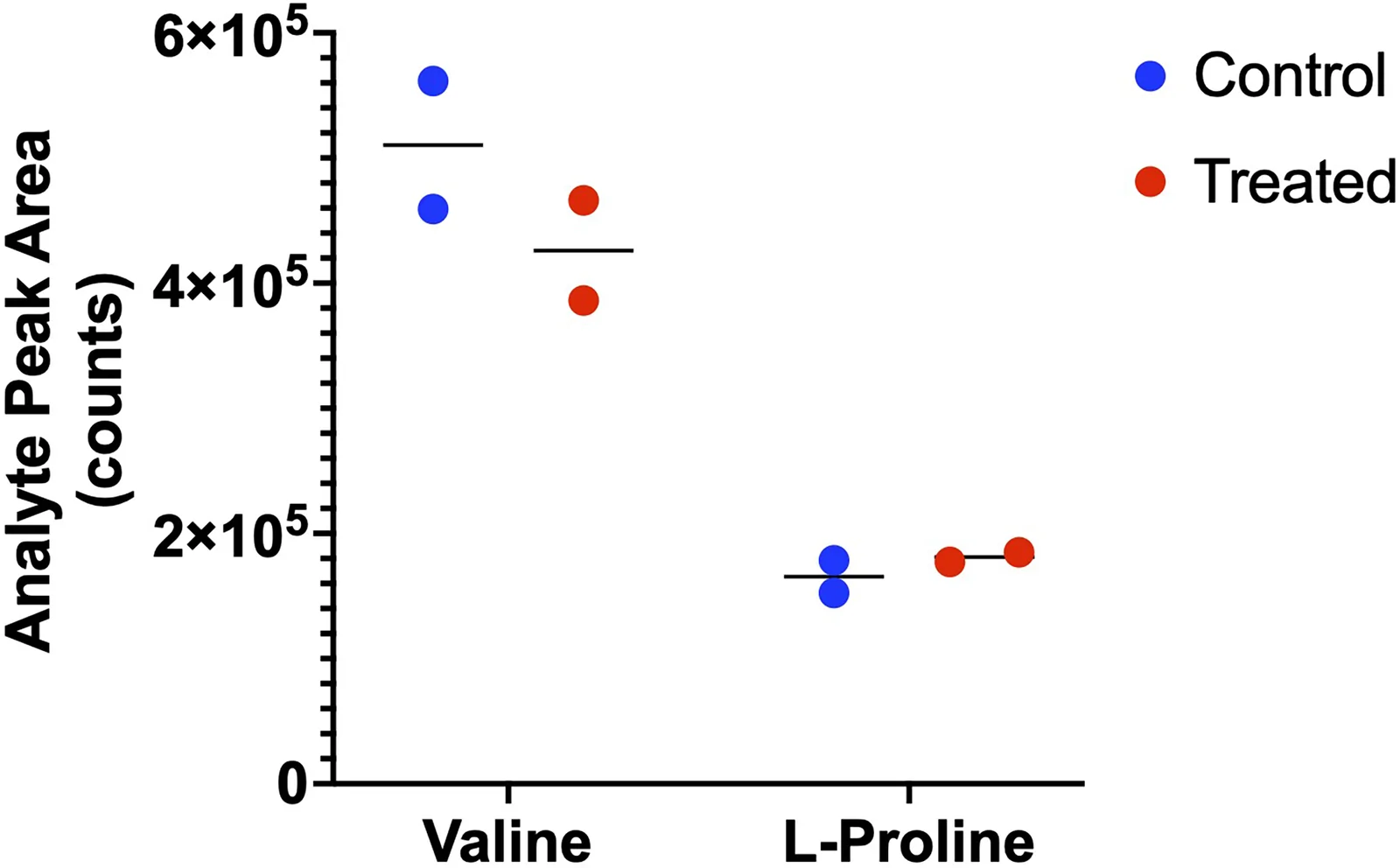
Effect of semapimod treatment on the intracellular levels of valine and L-proline in the Mtb mc^2^ 6206. Intracellular valine and L-proline were estimated in untreated (Control) and semapimod-treated (Treated) bacteria, after 24 hr of drug treatment, by mass spectrometry as described in Materials and methods. Mean + SD values from *n*=2 replicates are shown.

### Generation and characterization of semapimod-resistant strain of Mtb mc^2^ 6206

Semapimod-mediated inhibition of L-leucine uptake suggests the presence of a committed L-leucine transport machinery in mycobacteria. While a number of putative L-leucine transporter genes are found in *M. smegmatis* (Figure 4—figure supplement 1), none of their orthologues are present in Mtb.

**Figure 4.**
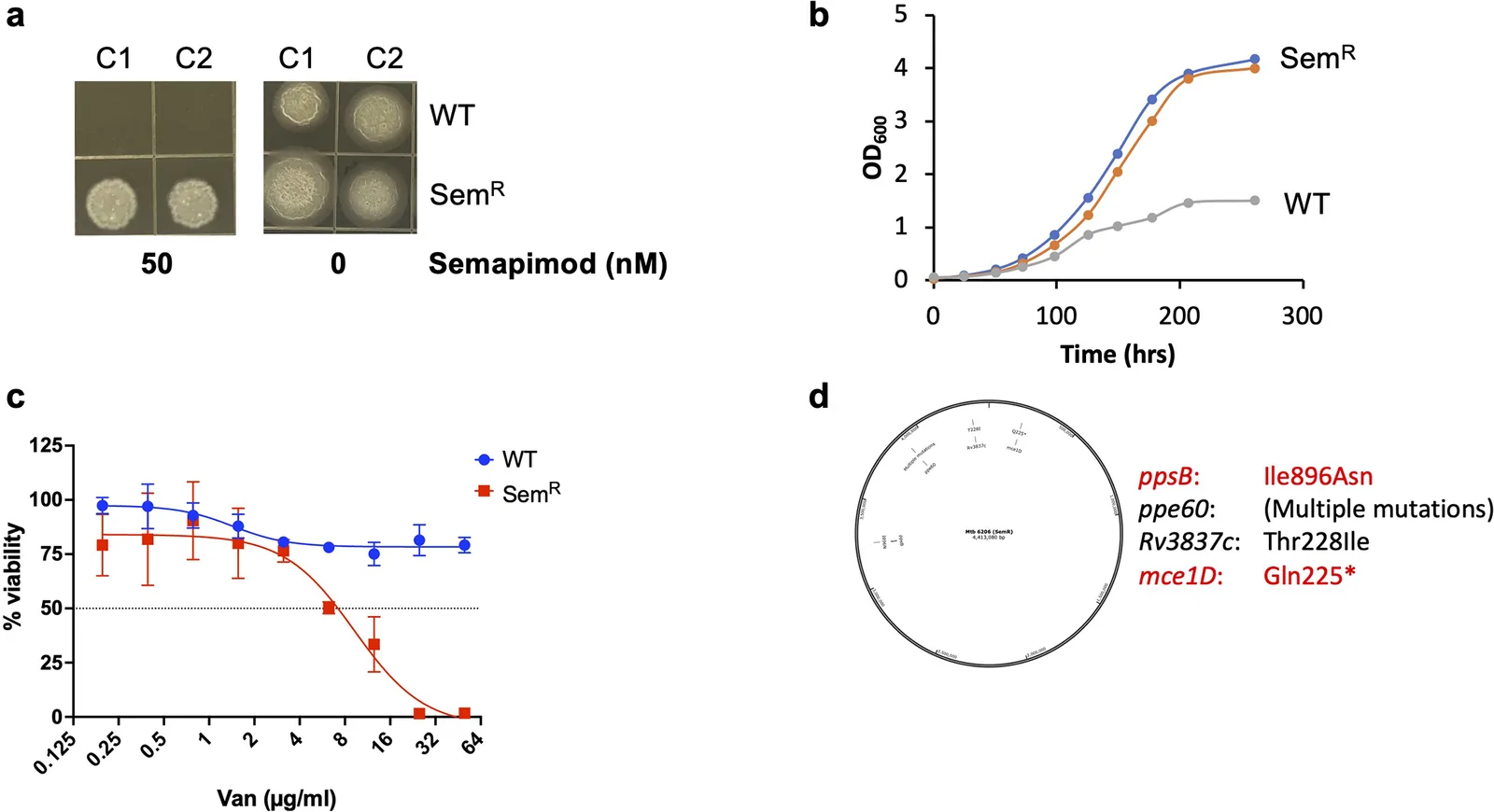
Generation and characterization of semapimod-resistant strain of Mtb mc^2^ 6206. (**a**) Propagation of the putative semapimod-resistant (Sem^R^) strain, but not the wild-type (WT) Mtb mc^2^ 6206 in the presence of 50 nM semapimod confirms drug-resistance in Sem^R^. In contrast, both the strains exhibit substantial growth on 7H11-PLO agar plate in the absence of drug. Shown is the growth pattern of two different colonies, C1 and C2, of the respective strains. (**b**) In vitro growth analysis of the WT and the Sem^R^ strains of Mtb mc^2^ 6206. Comparative analysis of OD_600_ at different time points reveals substantially increased growth of Sem^R^ strain compared to WT under in vitro culture conditions. (**c**) In vitro susceptibility of the WT and the Sem^R^ strains of Mtb mc^2^ 6206 to vancomycin. Viability of WT and Sem^R^ Mtb strains was determined in the presence of vancomycin by a 96-well plate-based assay, as described in Materials and methods. Percent viability was calculated with respect to the untreated (UT) cultures after 2 weeks of exposure to different concentrations of the antibiotic. Results show a substantial increase in the susceptibility of Sem^R^ to vancomycin compared to WT. (**d**) Whole-genome map analysis of Sem^R^. Shown is the genome map of Sem^R^ highlighting the positions of genes undergoing substitutions, when compared with WT Mtb mc^2^ 6206. Mutations leading to corresponding changes at the amino acid level in the respective proteins are indicated alongside. Suspect genes presumably involved in providing semapimod resistance are marked in red fonts. Data are representative of at least *n*=2 independent experiments in (**a**) and (**b**). Mean ± SD values from *n*=3 biological replicates are shown in (**c**). Figure 4—source data 1.Analysis of MIC_90_ of Rif and Inh against Semapimod^R^ strain of Mtb mc^2^ 6206.MIC_90_ of standard TB drugs, rifampicin, and INH was determined against WT and Sem^R^ strains using a 96- well plate- based assay, as described in the Materials and methods. As can be seen, resistance to semapimod does not alter bacterial susceptibility to either of the frontline TB drugs.

To gain insights into the potential mechanism of L-leucine transport in Mtb, we generated the semapimod-resistant (Sem^R^) strain of Mtb mc^2^ 6206 by repeated passaging in the presence of drug (Figure 4a). In vitro growth analysis reveals a significantly increased growth of the two independent Sem^R^ strains (Figure 4b). While the wild-type bacteria do not grow beyond OD_600_ of 2.0, both the Sem^R^ strains achieve an OD_600_ of ~4.0 after 10 days of culturing in 7H9-OADS medium supplemented with L-leucine and pantothenate (Figure 4b). Remarkably, intracellular L-leucine, but not the other BCAA such as valine, is significantly higher in the Sem^R^ strain when compared with the WT bacteria (Figure 4—figure supplement 2). The semapimod resistance does not lead to any change in susceptibility to standard TB drugs, such as rifampicin or isoniazid (Table 1); however, a marked increase in susceptibility to vancomycin is observed in the Sem^R^ bacteria. While vancomycin inhibits Sem^R^ with the IC_50_ of ~7 µg/mL, no effect is observed for this antibiotic against the wild-type Mtb mc^2^ 6206 (Figure 4c). It has been reported earlier that bacteria lacking PDIM grow faster and exhibit more susceptibility to vancomycin than the wild-type Mtb with intact PDIM, which provides a barrier to nutrients as well as large molecules such as vancomycin (Mulholland et al., 2024). Interestingly, whole-genome sequence analysis of the Sem^R^ strain reveals Ile896Asn mutation in PpsB, involved in PDIM synthesis (Figure 4d). In addition, three other genes show altered sequence in Sem^R^, namely, *mce1D*, *Rv3837c,* and *ppe60*. While *mce1D* accumulates a nonsense mutation yielding a stop codon at the 225th position, sequence alteration in *Rv3837c* is at the extreme 3′ end causing Thr → Ile substitution at the 228th position of the 332 amino acid long polypeptide. Furthermore, multiple mutations were observed exclusively in *ppe60* (Figure 4—figure supplement 3), despite the presence of two other paralogues of this gene (*ppe18* and *ppe19*), suggesting random accumulation of mutations in *ppe60* during passaging. These observations led us to investigate the association of *ppsB, mce1D,* and *ppe60* in semapimod resistance, whereas the possibility of involvement of *Rv3837c* is unlikely.

**Table 1.** Analysis of MIC_90_ of Rif and Inh against Semapimod^R^ strain of Mtb mc^2^ 6206. MIC_90_ of standard TB drugs, rifampicin, and INH was determined against WT and Sem^R^ strains using a 96-well plate-based assay, as described in the Materials and methods. As can be seen, resistance to semapimod does not alter bacterial susceptibility to either of the frontline TB drugs.

	Rifampicin	INH
**WT**	0.11 µM	0.40 µM
**Sem** ^**R**^	0.11 µM	0.40 µM

**Figure 4—figure supplement 1.**
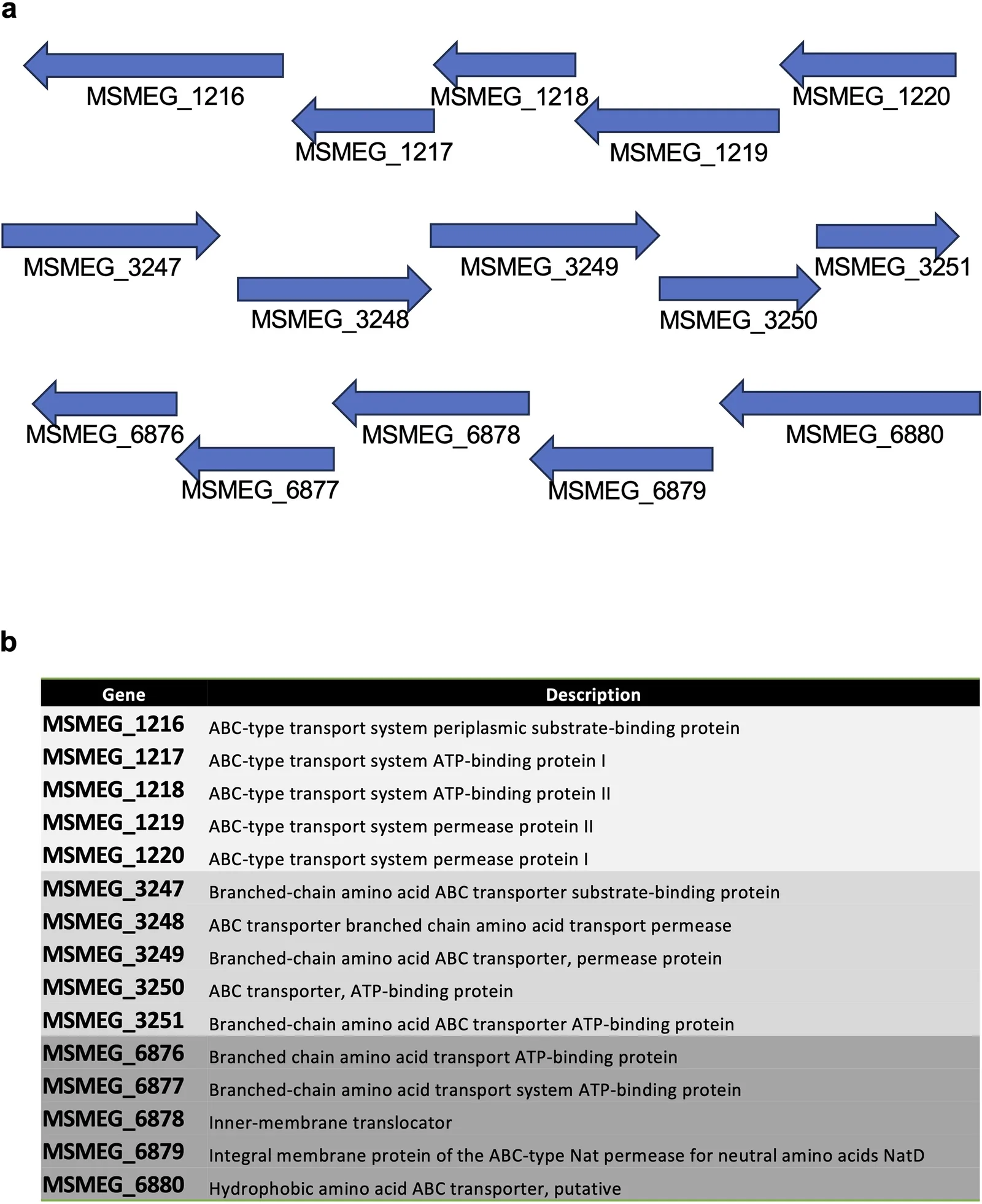
List of *M*. *smegmatis* genes potentially involved in L-leucine transport. (**a**) Organization of the prospective genes involved in L-leucine transport at different loci in *M. smegmatis*. (**b**) Description of the function of *M. smegmatis* genes associated with L-leucine transport.

**Figure 4—figure supplement 2.**
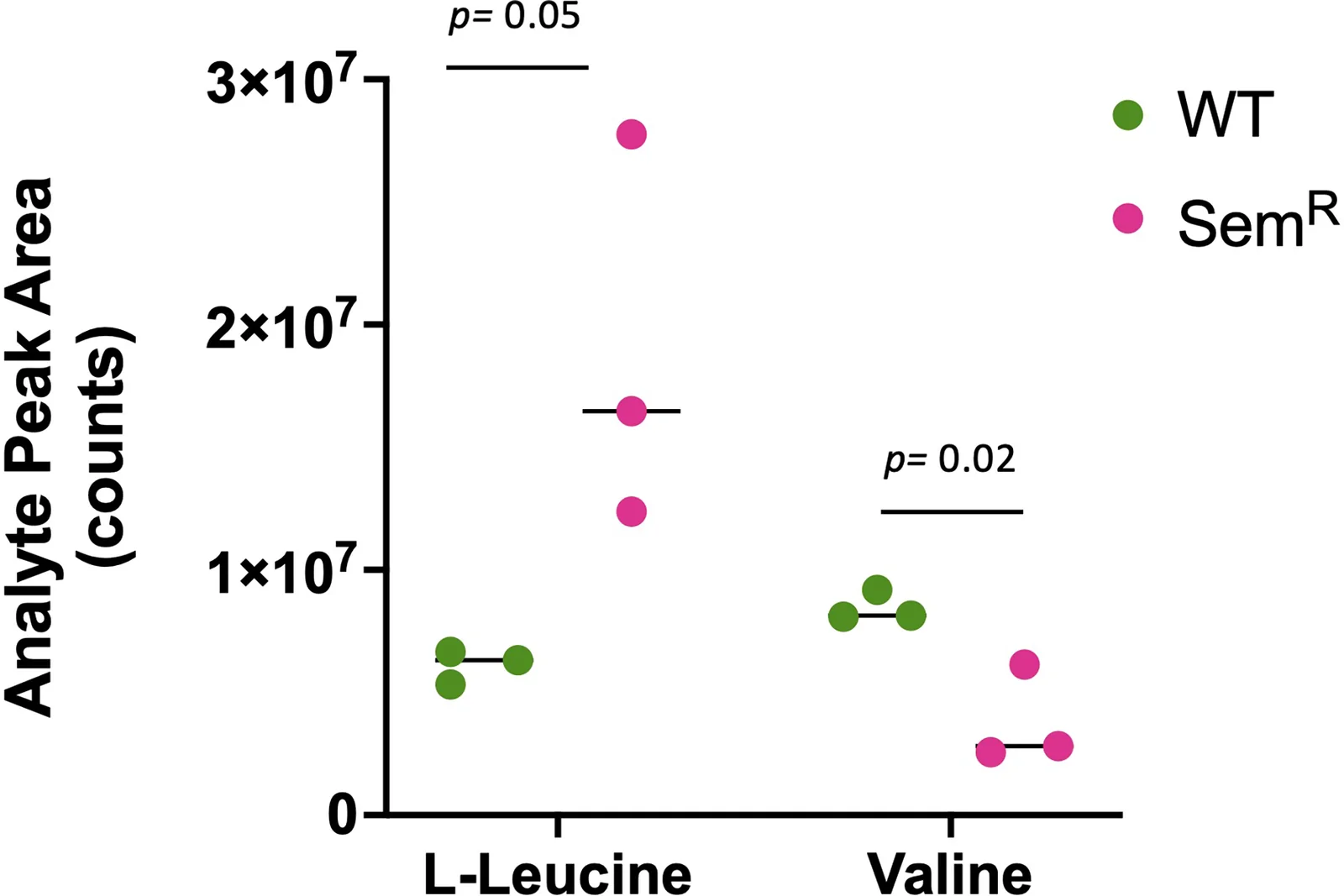
Estimation of intracellular L-leucine and valine in the Mtb mc^2^ 6206. Intracellular L-leucine and valine were estimated in the wild-type (WT) and Sem^R^ bacteria, after 7 days of culturing, by mass spectrometry as described in Materials and methods. Mean + SD values from *n*=3 replicates are shown. The p values were calculated using GraphPad Prism version 10.

**Figure 4—figure supplement 3.**
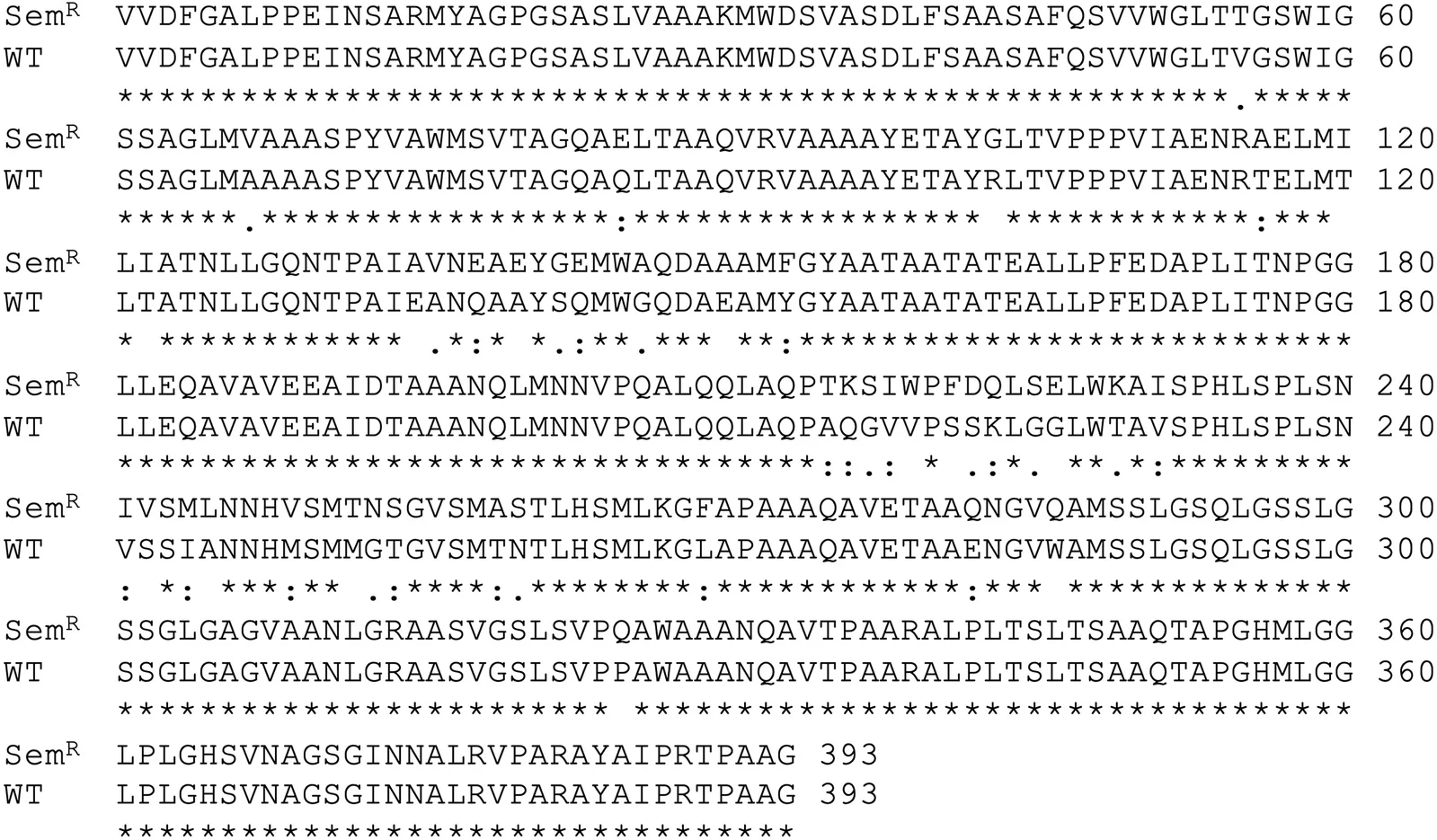
Comparative sequence analysis of Ppe60 in the wild-type and Sem^R^ strains of Mtb 6206. Sequence alignment of Ppe60 from WT and Sem^R^ strains was performed by Clustal Omega (https://www.ebi.ac.uk/jdispatcher/msa/clustalo) using default parameters. ‘*’ represents identical residues; ‘.’ represents weakly similar residues; ‘:’ signifies conserved residues with strongly similar properties, whereas completely dissimilar changes are shown by gaps.

### Overexpression of *ppsB* alters susceptibility of semapimod^R^ strain to drugs

**Figure 5.**
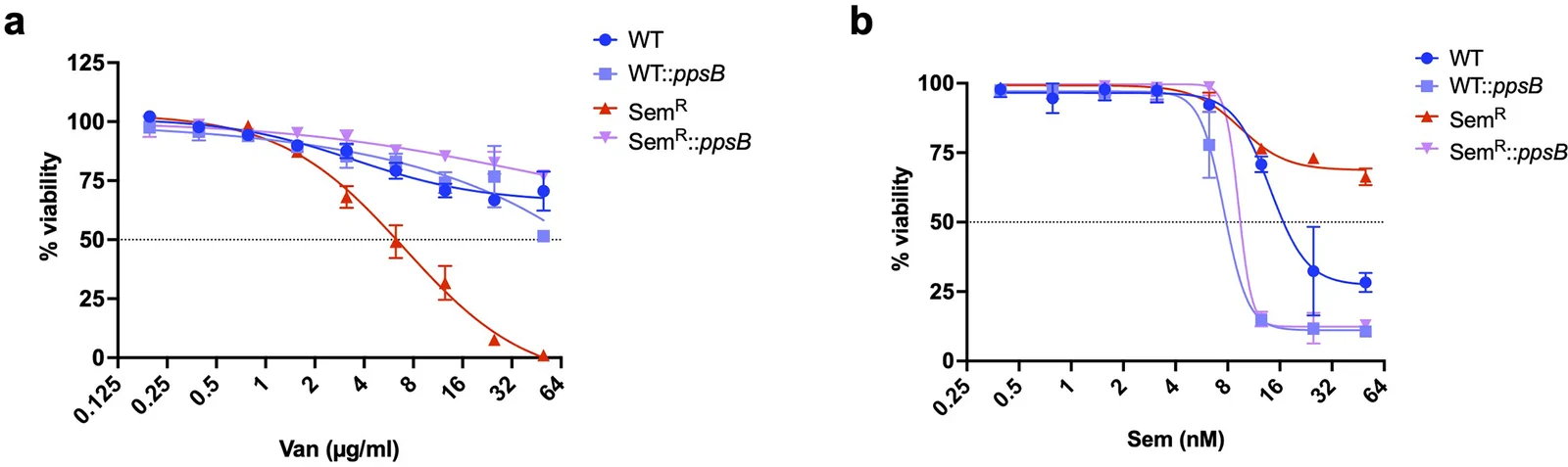
Overexpression of *ppsB* alters susceptibility of Sem^R^ strain of Mtb mc^2^ 6206 to semapimod and vancomycin. (**a–b**) In vitro susceptibility of different strains of Mtb mc^2^ 6206 to vancomycin (**a**) and semapimod (**b**). Viability of WT and Sem^R^ Mtb strains in the presence of drugs was compared with those constitutively expressing *ppsB* by a 96-well plate-based assay, as described in Materials and methods. Percent viability was calculated with respect to UT cultures after 2 weeks of exposure to different concentrations of inhibitors. Remarkably, the response of Sem^R^ to both semapimod and vancomycin is reversed upon overexpression of *ppsB*. Contrarily, *ppsB* expression in WT does not affect bacterial sensitivity to either of these drugs, thus indicating a specific effect of the PDIM biosynthesis gene in Sem^R^. Mean values from *n*=3 biological replicates are shown in (**a**) and (**b**).

To further identify genes that might contribute to semapimod resistance, we overexpressed *ppsB, mce1D,* and *ppe60* genes in the wild-type and Sem^R^ strains of Mtb mc^2^ 6206 under the constitutive promoter. Notably, susceptibility to vancomycin and semapimod was completely reversed in Sem^R^ strain upon overexpression of *ppsB*, whereas no effect of *ppsB* overexpression is observed in the wild-type bacteria. Sem^R^ strain, which is susceptible to vancomycin, becomes resistant akin to the wild-type strain by overexpression of *ppsB* (Figure 5a). Similarly, susceptibility to semapimod is restored in Sem^R^ by overexpressing *ppsB* (Figure 5b). In contrast, no effect of *mce1D* or *ppe60* overexpression is observed on bacterial sensitivity to semapimod (Figure 5—figure supplements 1 and 2).

**Figure 5—figure supplement 1.**
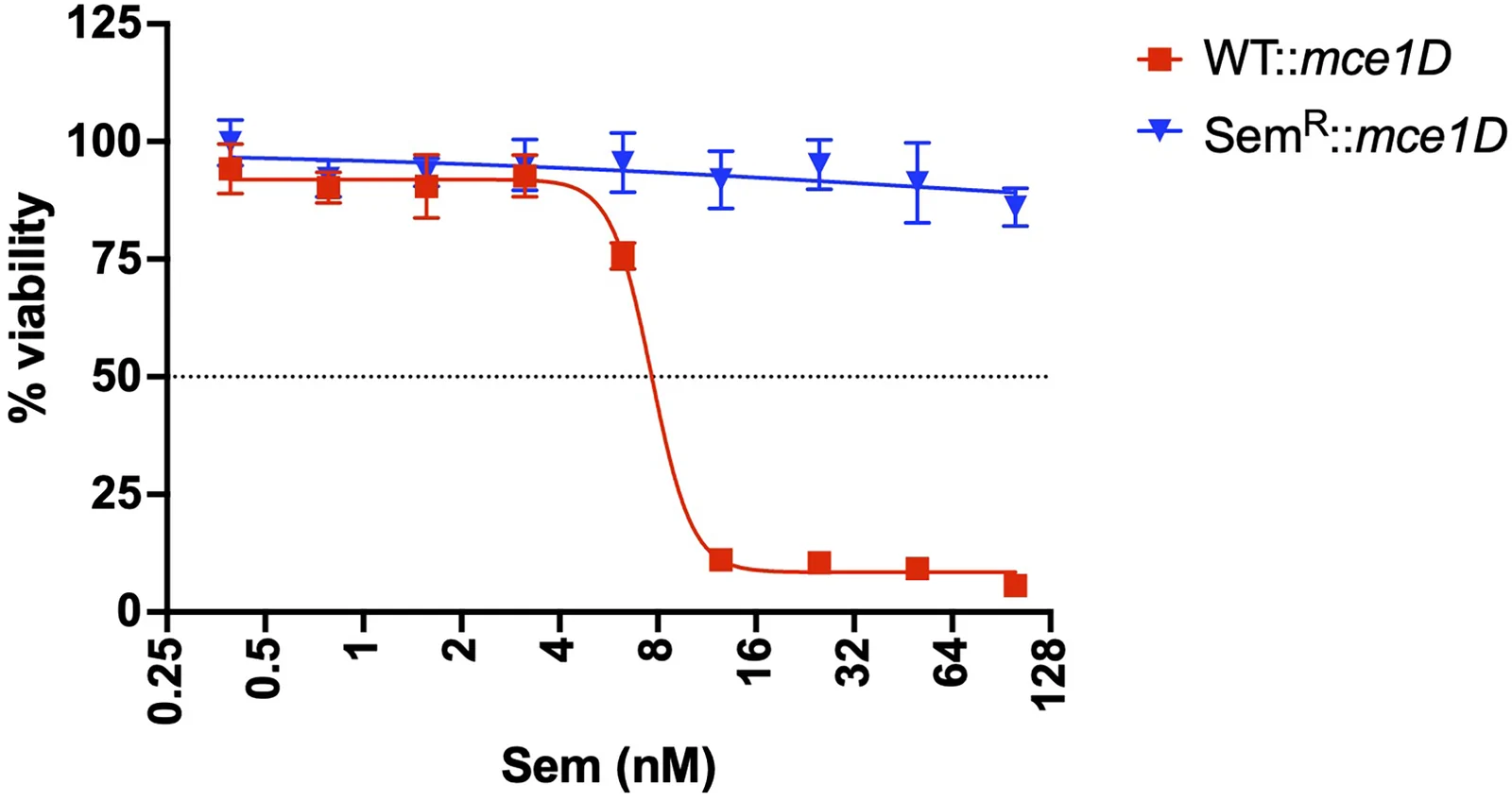
Overexpression of *mce1D* does not affect susceptibility of Mtb mc^2^ 6206 to semapimod. Effect of *mce1D* overexpression in the WT and Sem^R^ strains on bacterial viability in the presence of semapimod. Viability of WT and Sem^R^ strains constitutively expressing *mce1D* was assessed by a 96-well plate-based assay, as described in Materials and methods. Percent viability was calculated with respect to UT cultures after 2 weeks of exposure to different concentrations of inhibitors. Mean ± SD values of *n*=2 biological and *n*=2 technical replicates are shown.

**Figure 5—figure supplement 2.**
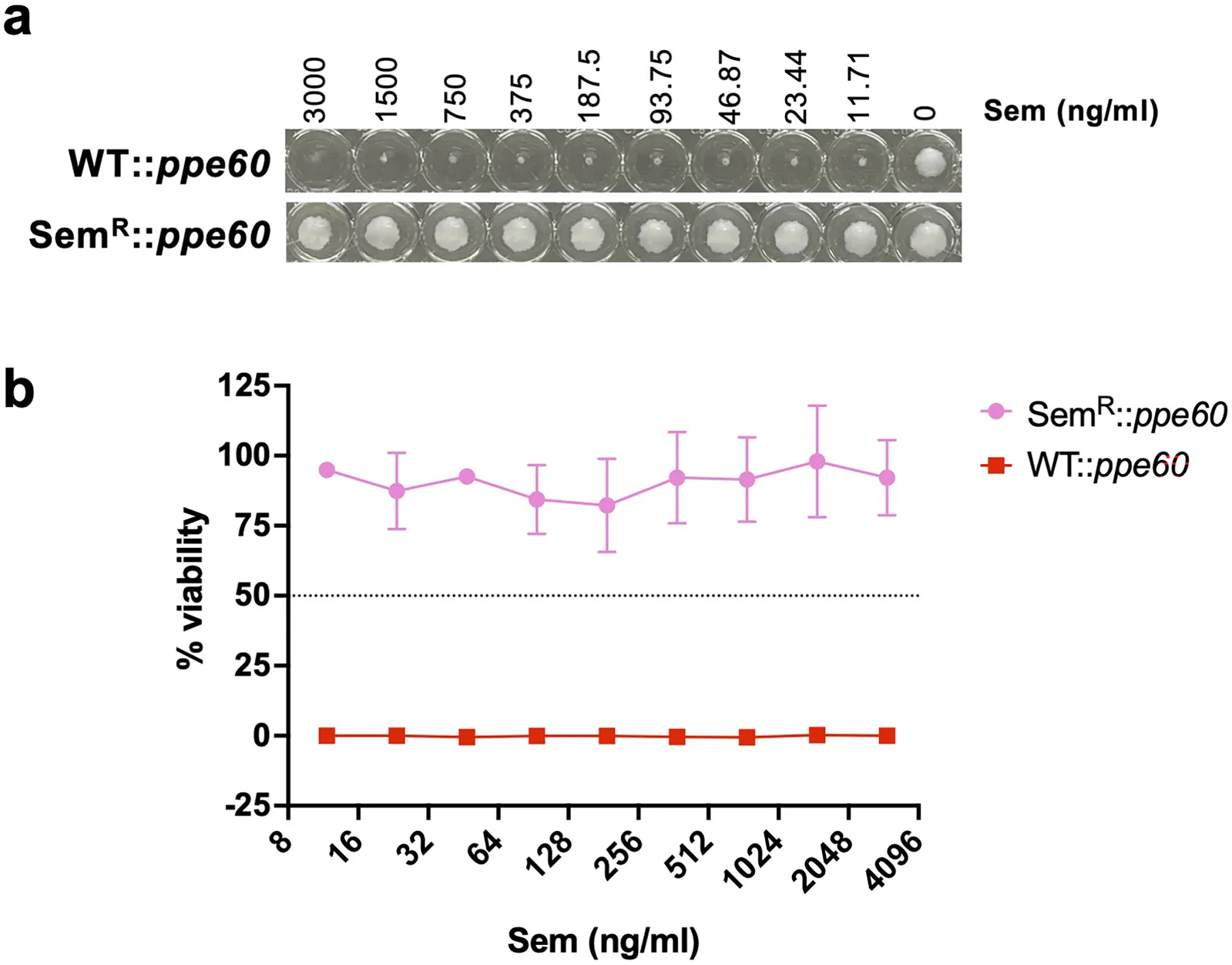
Overexpression of *ppe60* does not affect susceptibility of Mtb mc^2^ 6206 to semapimod. (**a–b**) Effect of *ppe60* overexpression in the WT and Sem^R^ strains on bacterial viability was determined in the presence of semapimod by a 96-well plate-based assay, as described in Materials and methods. Viability was assessed by visual inspection of the plate (**a**) as well as by determining percent viability (**b**). Percent viability was calculated with respect to UT cultures after 2 weeks of exposure to different concentrations of semapimod. Mean ± SD values of *n*=2 biological replicates are shown.

### Semapimod targets PpsB of *M. tuberculosis*

**Figure 6.**
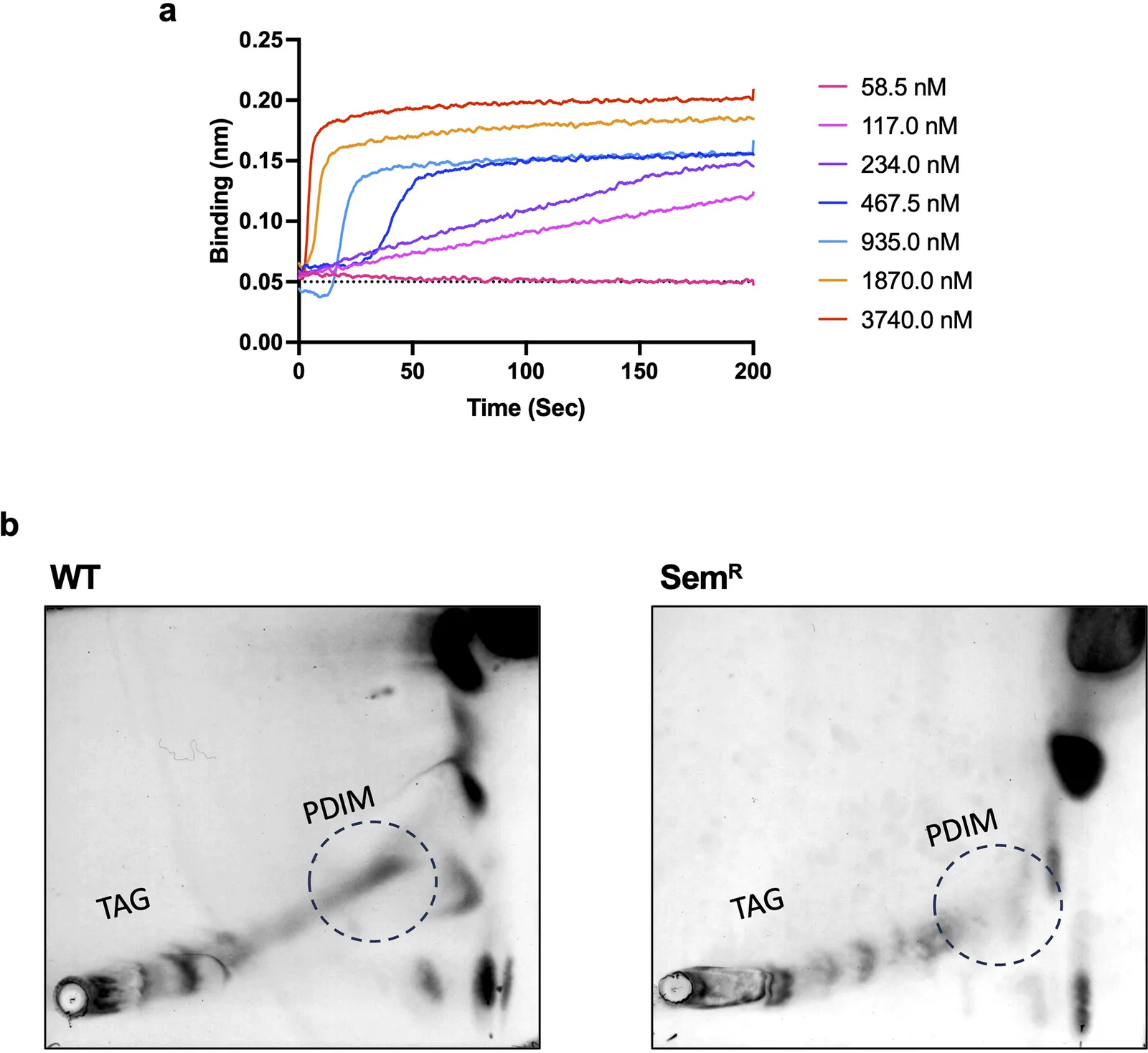
Semapimod targets the PDIM biosynthesis protein, PpsB. (**a**) Analysis of semapimod-PpsB interaction by BLI-Octet. Interaction of semapimod with Mtb PpsB was examined by optical interference-based biolayer interferometry from the Octet system (ForteBIO). Briefly, dialyzed 6xHis-PpsB was immobilized onto an AR2G sensor to a level of 2.1 nm. Binding was observed at the indicated concentrations of the ligand (semapimod) to acquire a differential graded response. The binding constant was calculated as per the standard steps described in Materials and methods. (**b**) Analysis of cell-wall apolar lipids in the WT and Sem^R^ strains of Mtb mc^2^ 6206. Cell-wall apolar lipids were extracted and analyzed by two-dimensional TLC as described in Materials and methods, revealing a significant reduction in PDIMs in Sem^R^ compared with the WT strain. Contrary to PDIMs, no change was observed in the triacylglycerol (TAG) levels between the two strains. The position of PDIMs is marked by broken circles in both images for clarity. Figure 6—source data 1.PDF file containing original TLC images for Figure 6B, indicating the positions of TAG and PDIM in Sem^R^ and WT strains. Figure 6—source data 2.Original file for TLC images displayed in Figure 6B.

The above results strongly suggest that the cell-wall PDIM biosynthesis protein PpsB is involved in semapimod-mediated interference of L-leucine uptake in Mtb. To corroborate our findings, we sought to examine if PpsB of Mtb is directly targeted by semapimod. The 4617 bp *ppsB* open-reading frame (ORF) of Mtb was cloned in an *E. coli* expression plasmid, pET28, for overexpression and purification of 6x His-tagged PpsB of Mtb. Analysis of purified protein by SDS-PAGE reveals near-homogeneous preparation of the protein migrating at its predicted molecular mass of ~170 kDa (Figure 6—figure supplement 1). The purified 6x His-PpsB was then subjected to interaction with different concentrations of semapimod using Biolayer Interferometry-Octet system (Sartorius), as mentioned in the Materials and methods. The results, presented in Figure 6a, clearly demonstrate a dose-dependent interaction of semapimod with PpsB, immobilized on AR2G sensor. Further analysis of binding kinetics reveals that semapimod strongly binds to PpsB with a dissociation constant (*K_d_*) of 110 nM. As a control, interaction of semapimod was also analyzed with the purified Ppe60, which fails to exhibit any binding.

**Figure 6—figure supplement 1.**
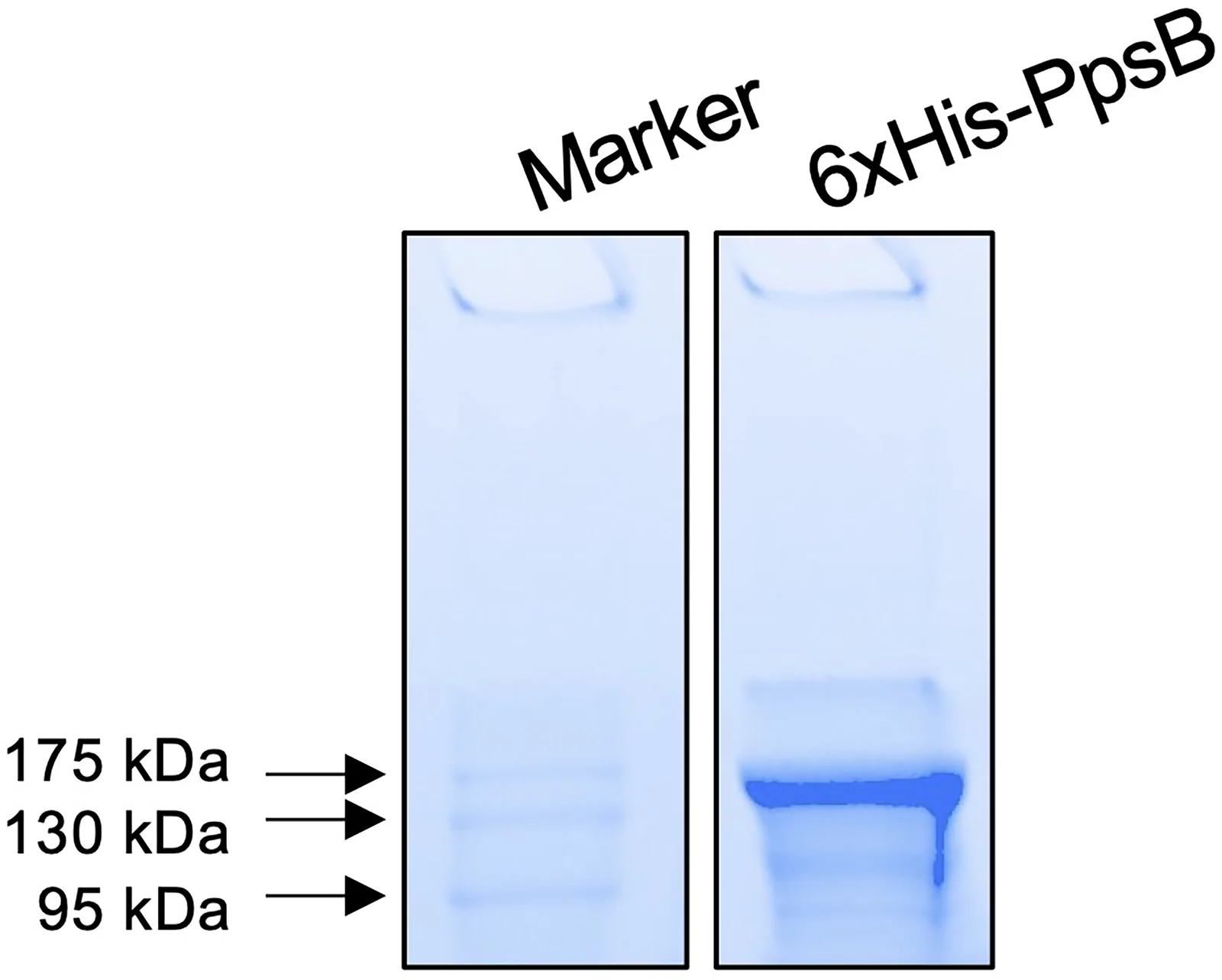
SDS-PAGE analysis of purified PpsB of Mtb. Shown is the Coomassie Brilliant Blue-stained denaturing polyacrylamide gel with 10 µg of purified PpsB with 6 x His tags at the N-terminus. The molecular mass of the purified protein was determined by using the prestained molecular mass marker. Figure 6—figure supplement 1—source data 1.PDF file containing original Coomassie-stained polyacrylamide gel for Figure 6—figure supplement 1 showing the elution fractions of purified PpsB. Figure 6—figure supplement 1—source data 2.Original file for Coomassie-stained polyacrylamide gel displayed in Figure 6—figure supplement 1.

**Figure 6—figure supplement 2.**
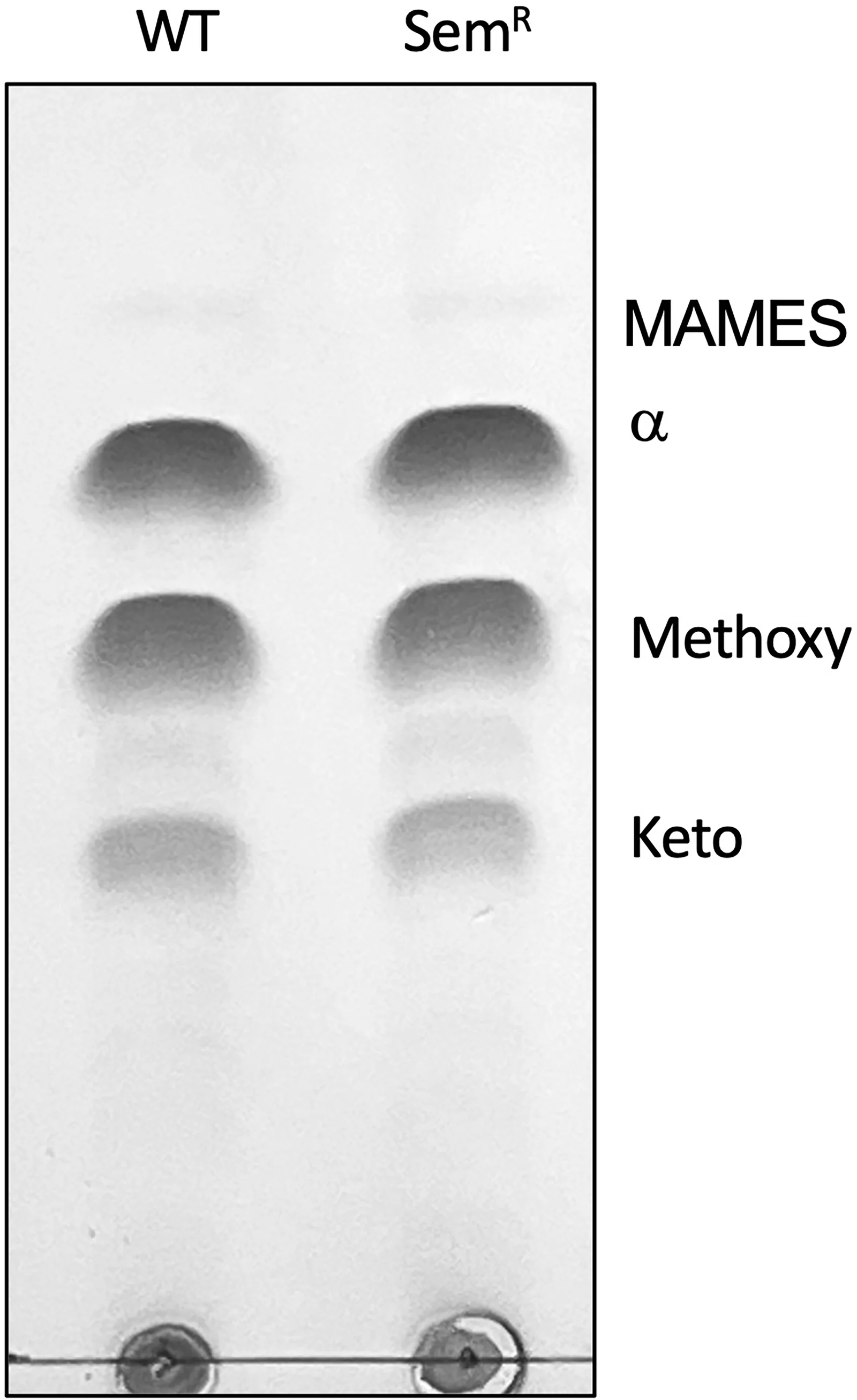
Comparative analysis of cell-wall mycolic acid profile of WT and Sem^R^ strains of Mtb mc^2^ 6206. Cell-wall mycolic acid methyl esters (MAMES) were extracted from both strains and analyzed by one-dimensional TLC, as described in Materials and methods. As can be seen, no change was observed in the levels of different MAMES between the two strains. Figure 6—figure supplement 2—source data 1.PDF file containing original TLC image for Figure 6—figure supplement 2 showing MAMES in Sem^R^ and WT strains. Figure 6—figure supplement 2—source data 2.Original file for TLC image displayed in Figure 6—figure supplement 2.

### PDIM is compromised in Sem^R^ strain

As hitherto mentioned, PDIM has been found to act as a barrier for the uptake of nutrients and large-size inhibitors. Moreover, our results clearly establish that semapimod targets PDIM biosynthesis protein PpsB by direct binding. Based on our findings, we speculate that semapimod resistance is acquired in Mtb owing to disruption in the cell-wall PDIM, which alleviates the regulation of L-leucine uptake. To test our hypothesis, we analyzed the cell-wall lipids of the wild-type and Sem^R^ strains of Mtb mc^2^ 6206. Both mycolic acids and PDIM were extracted from these strains and analyzed by thin-layer chromatography (TLC), as described in the Materials and methods. While no difference in the profiles of mycolic acid methyl esters or the triacylglycerol (TAG) was noticed between the two strains, the Sem^R^ exhibits a marked reduction in PDIM (Figure 6b, Figure 6—figure supplement 2).

### Intracellular survival of Mtb H_37_Rv pathogen in the organelles of infected BalB/C mice is decreased upon semapimod treatment

**Figure 7.**
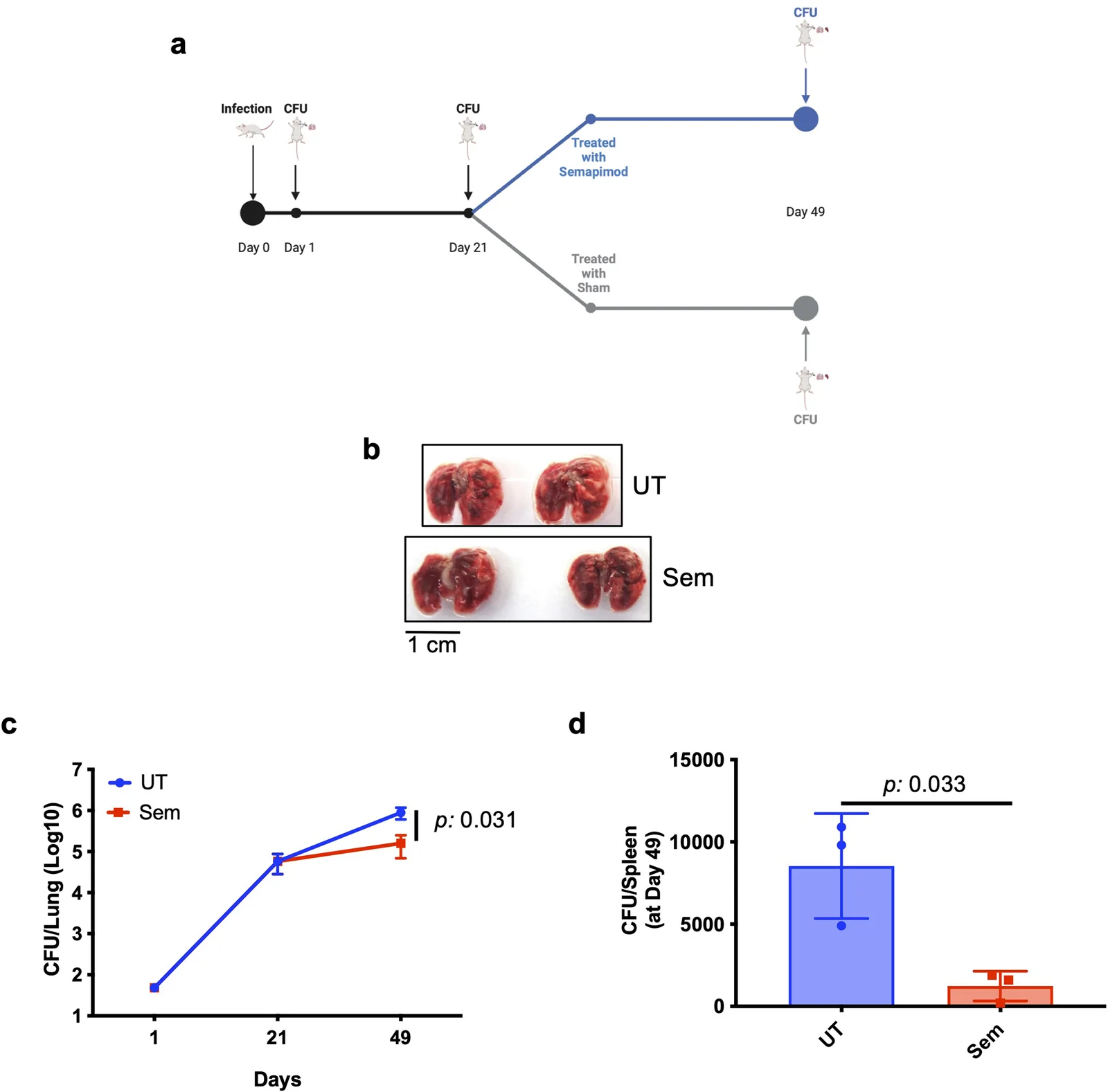
Effect of semapimod treatment on the survival of Mtb H37Rv in the organs of infected BALB/c mice. (**a**) Schematic of mouse infection. Infection was performed by the aerosol route with the virulent Mtb H_37_Rv strain. After 21 days of infection, mice were divided into two groups: one receiving only 5% sucrose (sham) and the other receiving 5 mg/kg semapimod prepared in 5% sucrose. Intracellular bacterial load was determined by CFU plating of lung homogenates at days 1, 21, and 49, and of spleen homogenates prepared on day 49. (**b**) Gross pathology of lungs. Images of lungs obtained from both the sham- and semapimod-treated groups of mice after 28 days of treatment (i.e. day 49 post-infection) are presented. Scale bar is shown for size reference. (**c–d**) Effect of semapimod treatment on intracellular survival of Mtb H_37_Rv. Intracellular survival was determined by estimating the bacterial burden in the lungs at the respective time points (**c**), and in the spleen at day 49 post-infection (**d**), by CFU enumeration. Data represent mean ± SD values from *n*=3 animals in (**c**) and (**d**). p Values in (**c**) and (**d**) were obtained after comparison of CFUs between the two groups, as described in Materials and methods. The illustration was created in BioRender.

Although endogenous leucine biosynthesis is extremely essential for bacterial survival and virulence, there is no study describing the underlying mechanism of L-leucine uptake from the extracellular environment and its importance in mycobacterial virulence during animal infections. To examine if L-leucine uptake is critical for the intracellular survival of the pathogenic Mtb H_37_Rv strain with intact endogenous leucine biosynthesis, we tested the effect of semapimod treatment on the intracellular burden of Mtb H_37_Rv during mouse infection. Infection was performed via aerosol route as described in the Materials and Methods. After 21 days of infection, a group of three mice was treated with a 5 mg/kg dose of semapimod, whereas another group of three mice was treated with vehicle (1 x PBS) by daily oral gavaging for 4 weeks, typically as shown in the schematic. Bacterial load was examined in the lungs and spleen of animals from both groups by CFU estimation (Figure 7a). Examination of the gross lung pathology reveals a large number of granulomatous nodules in the untreated infected group, whereas semapimod treatment led to a significant reduction in the disease pathology (Figure 7b). Remarkably, semapimod treatment caused ~82% and ~85% reduction in CFU counts in the lungs and spleen, respectively, after 4 weeks of semapimod treatment (Figure 7c and d). Noteworthy to mention, in vitro susceptibility to vancomycin remained unaffected in bacteria retrieved from the lungs of the untreated and semapimod-treated mice (Figure 7—figure supplement 1), thus indicating comparable levels of cell wall PDIMs, one of the virulence factors, in both untreated and semapimod-exposed Mtb H_37_Rv.

**Figure 7—figure supplement 1.**
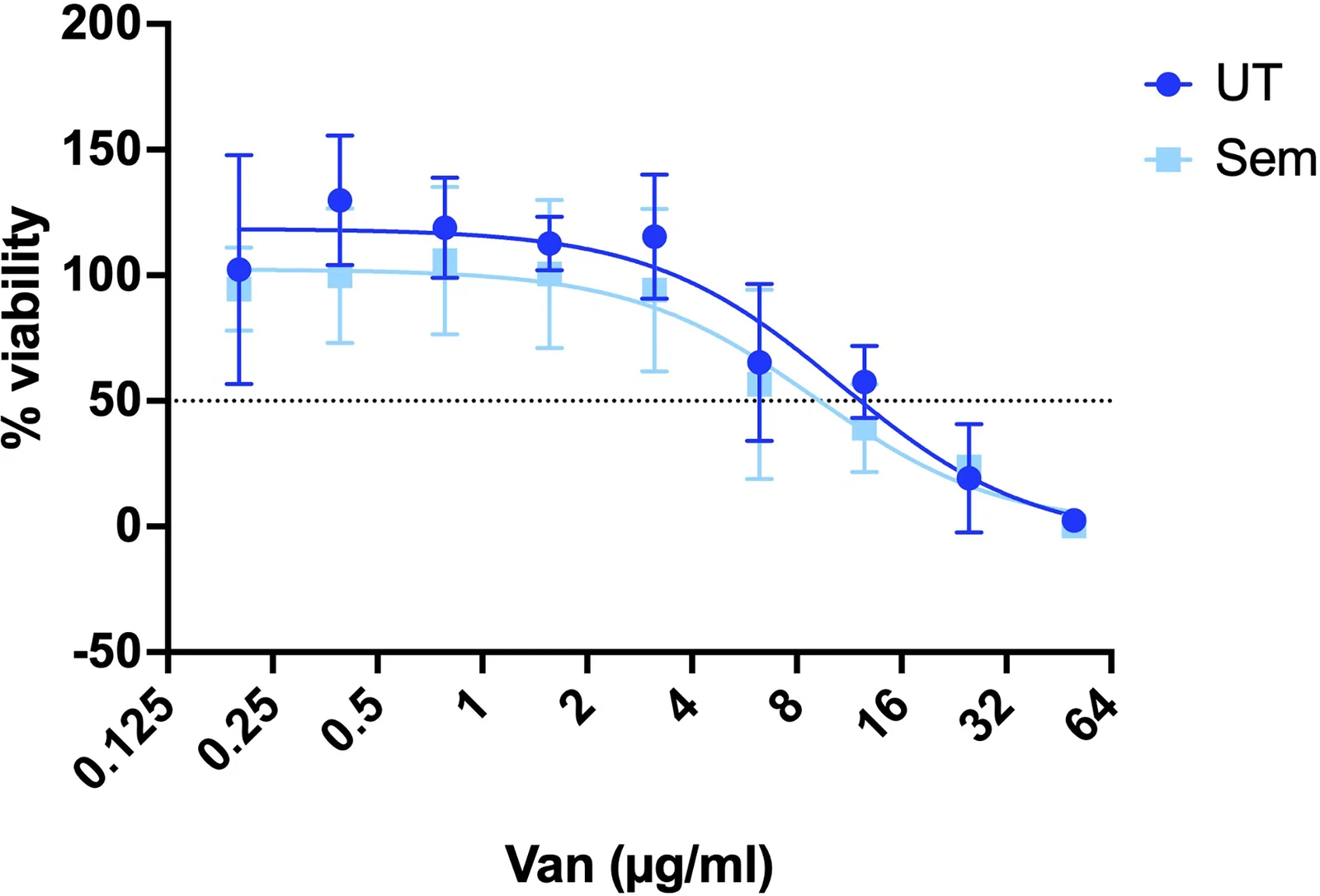
Susceptibility of Mtb H37Rv isolated from mice lungs post-semapimod treatment to vancomycin. Susceptibility of Mtb H37Rv obtained from the lungs of untreated (UT) and semapimod-treated (Sem) infected mice to vancomycin was assessed by a 96-well plate-based assay, as described in Materials and methods. Percent viability was calculated with respect to UT cultures after 2 weeks of exposure to different concentrations of vancomycin. Mean ± SD values of *n*=2 biological replicates are shown.

Overall, these results provide first evidence of the effect of a repurposed compound obstructing the in vitro L-leucine uptake on Mtb virulence and TB pathogenesis during infection.

## Discussion

Conventional approaches of drug discovery involve screening of NCEs against either the validated ‘drug target’ (target-based screen) or the pathogen as a whole (phenotypic screen; Rütten et al., 2022). The aim is to identify inhibitor(s) showing a novel MoA. However, any promising NCE faces a multitude of barriers to reach market launch from initial development (Payne et al., 2007). Repurposing offers the possibility of identifying new MoA of the existing drugs that are already approved for human use (Strittmatter, 2014; Tobinick, 2009). Therefore, it bypasses the traditional drug discovery and development pipeline involving de novo synthesis of NCEs. Several non-antibiotic drugs that are currently approved or under development may inhibit microbial pathogens, and therefore can be explored as novel antimicrobials, either alone or in combination with the existing antibiotics (Koh Jing Jie et al., 2022; Temel and Aksoyalp, 2024).

The present study was designed with the aim to identify novel TB inhibitors using the FDA-approved repurposed library comprising over 3600 molecules. Remarkably, in addition to the known anti-TB drugs, we found several novel hit molecules that were developed against non-communicable diseases such as cancer, cardiovascular, metabolic, and neurological diseases (Figure 1a–b) that can be explored as novel anti-TB drugs in the future. Notably, one of the top hits, namely semapimod, which inhibited Mtb mc^2^ 6206 growth at a concentration of less than 20 nM, has never been reported to have antibiotic activity. We show that this drug not only kills Mtb mc2 6206 in vitro, but also significantly reduces bacterial growth during macrophage infection (Figure 1c–e). Surprisingly, semapimod did not exhibit growth inhibition of other mycobacteria including the pathogenic Mtb H37Rv (Figure 3), which was used to develop the auxotroph by deletion of *leuC-leuD* and *panC-panD* genes. Remarkably, we demonstrate that growth inhibition by semapimod is due to blockage of L-leucine availability in Mtb mc^2^ 6206 (Figure 3). We rule out the possibility of L-leucine quenching or modification by semapimod, as both the *leuC-leuD*-expressing and the Sem^R^ strains of Mtb mc^2^ 6206 continue to metabolize exogenous L-leucine in the presence of drug. Instead, our results suggest that semapimod restricts L-leucine uptake in Mtb. As a consequence, this drug is not effective against other mycobacterial species with the functional endogenous leucine biosynthesis pathway.

**Figure 8.**
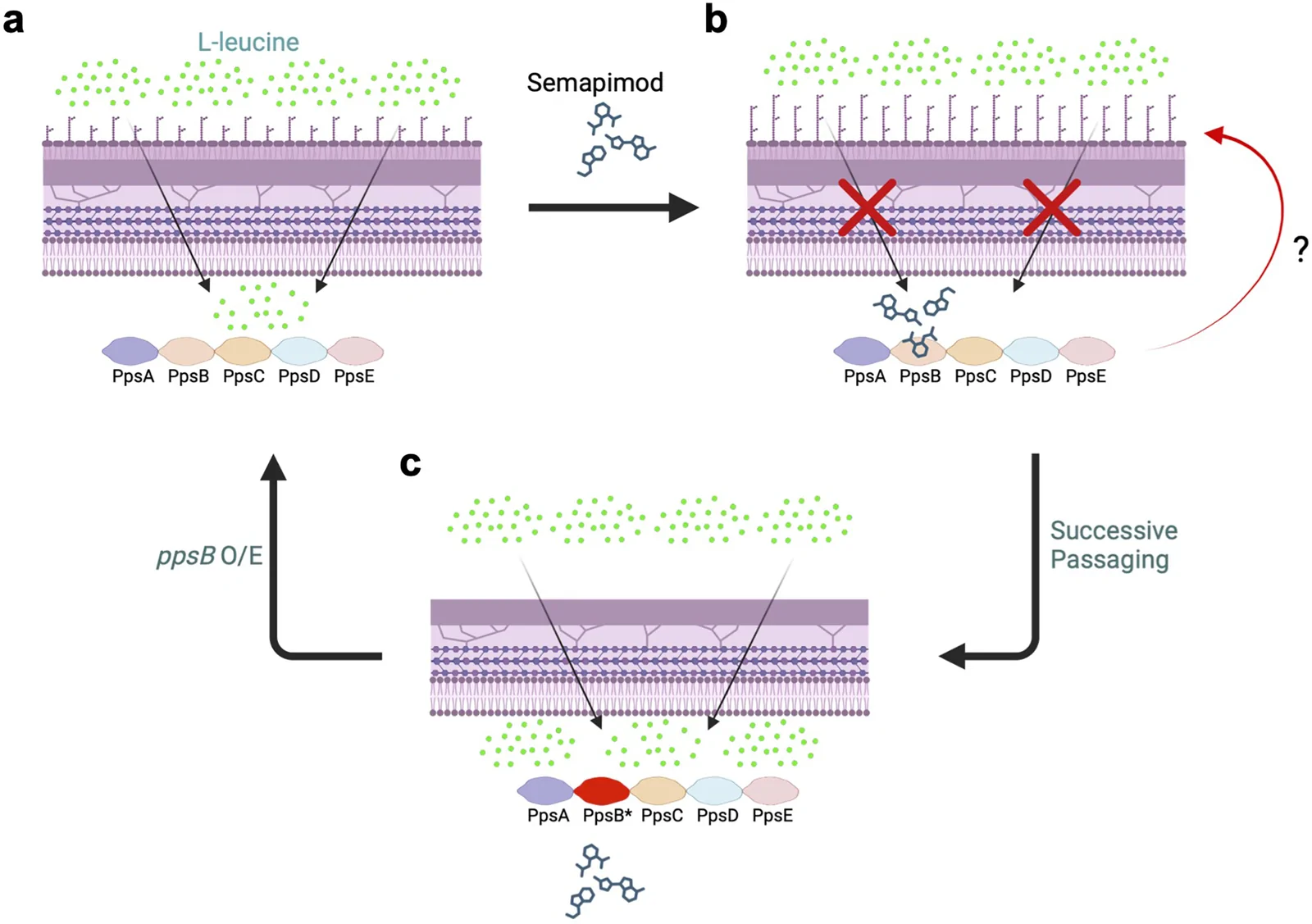
Proposed model describing the effect of semapimod on L-leucine uptake in Mtb mc^2^ 6206. (**a**) Uptake of L-leucine is controlled by cell-wall PDIM. (**b**) Semapimod targets the PDIM biosynthesis protein (PpsB) causing an altered PDIM profile, which restricts L-leucine uptake in the auxotroph by an unknown mechanism (denoted by ‘?’), leading to cell death. (**c**) L-leucine is freely accessible to the Sem^R^ strain, owing to perturbation of cell-wall PDIM. As a consequence, semapimod is ineffective against this strain. However, overexpression of wild-type *ppsB* in Sem^R^ reverts its susceptibility to semapimod. The illustration was created in BioRender.

Cell membrane acts as a permeability barrier to foreign substances and selectively filters molecules that are essential for cellular physiology. Transport of solutes involves three different classes of transport mechanisms, viz., passive diffusion, facilitated diffusion, and active transport. Of these three different classes, amino acid transport fulfills the criteria of facilitated diffusion and active transport (Quigley, 2017). The two best characterized amino acid transport systems are the high-affinity periplasmic binding protein-(BP) dependent transport system for leucine, isoleucine, and valine (LIV-I and LIV-II) in *E. coli* and the high-affinity histidine transport system in *Salmonella typhimurium (*Haney and Oxender, 1992; Antonucci et al., 1985; Landick and Oxender, 1985; Guardiola et al., 1974; Landick et al., 1985). Although Mtb is able to synthesize all 20 amino acids (Yelamanchi and Surolia, 2021; Prakash et al., 2005; Giffin et al., 2012), to ensure a constant supply of amino acids under a wide variety of environments, the TB pathogen must be equipped for their transport. Genome analysis of the fast-growing soil organism, *M. smegmatis* reveals 15 genes spread across three different loci that are putatively involved in BCAA transport (Figure 4—figure supplement 1). However, homologues of none of these are found in Mtb. Although intracellular Mtb exploits multiple host-derived amino acids as nitrogen sources, including BCAAs, during growth in macrophages (Borah et al., 2019; Gouzy et al., 2014), it remains unclear how amino acids are transported across the cell envelope of Mtb. To the best of our knowledge, this is the first study providing evidence for the presence of a functional transport system for a BCAA in Mtb. We show that semapimod, which is an anti-inflammatory drug, targets a polyketide synthase (PKS) involved in the cell-wall PDIM biosynthesis, and selectively kills in vitro the leucine auxotroph by blocking the L-leucine uptake, possibly by modulating the PDIM architecture. The cell-wall PDIM regulates the uptake of nutrients and provides a barrier to large molecule inhibitors. PDIM tends to shed during culturing of Mtb in the presence of ionic detergents, thus providing better access to nutrients and large molecules such as vancomycin. As a consequence, Mtb strains lacking PDIM not only grow faster but also become more susceptible to vancomycin (Mulholland et al., 2024). Remarkably, the Sem^R^ strain which accumulates mutation in the PpsB near acetyltransferase domain exhibits both the phenotypes, that is, a dramatic increase in its susceptibility to vancomycin and faster growth rate compared to the wild-type Mtb, despite culturing in the presence of the non-ionic detergent. Loss of PDIM in the drug-resistant strain is further confirmed by TLC analysis of the PDIM, whereas other lipids such as TAG or mycolic acids remain unaltered (Figure 6b, Figure 6—figure supplement 2). Most importantly, susceptibility to semapimod is restored in the Sem^R^ Mtb mc^2^ 6206 by overexpression of *ppsB*. Taken together, these results clearly demonstrate for the first time the involvement of the cell-wall PDIM in L-leucine uptake in Mtb (Figure 8). At present, it is not understood how PDIM selectively facilitates the movement of L-leucine, and whether other apolar hydrophobic molecules follow the same route for their transport across the mycobacterial cell membrane. Since PDIM is an important virulence factor (Rens et al., 2021), understanding its role in the transport of other metabolites including amino acids will shed important light on virulence strategies employed by Mtb.

Semapimod treatment interferes with the expression of more than 10% of protein-coding genes of Mtb involved in a variety of functions. A careful analysis of the transcriptional profile further provides evidence corroborating the deviation of leucine levels in the cell by semapimod treatment. For instance, *lrpA*, which encodes for a transcriptional regulator of the leucine-responsive regulatory protein (Lrp) family (Song et al., 2016), is upregulated by 9.25-fold. It has been shown that binding of Mtb LrpA with the target promoters is influenced by amino acids and vitamins (Song et al., 2016). Not only LrpA, but also some of its regulons such as *lat* (94.01-fold), *whiB2* (0.33-fold), *pks2* (12.58-fold), and *gid* (0.37-fold) also show significant modulation.

Among other differentially expressed gene sets, the most notable ones are those involved in energy and lipid metabolism. Since leucine is catalyzed into acetyl-CoA (Massey et al., 1976), we hypothesize that in the presence of semapimod, the level of acetyl-CoA is perturbed in the cell. Acetyl-CoA, being the precursor of energy metabolism, subsequently impacts the overall energy state, as evidenced by >80% reduction in ATP following drug treatment. In other bacteria, such as *Salmonella*, L-leucine supplementation stimulates the TCA cycle, causing increased intracellular NADH and ATP concentrations (Yang et al., 2023), which further substantiates the contribution of intracellular leucine to energy production in mycobacteria.

Strikingly, semapimod-treated Mtb exhibits significant upregulation of genes that encode for proteins involved in MCC such as methylcitrate synthase PrpC (19.48-fold), methylcitrate dehydratase PrpD (92.5-fold), the regulator of *prpC* and *prpD* genes, PrpR (28.55-fold), and an enzyme, Icl1 (68.69-fold). Icl1 of Mtb exhibits both isocitrate lyase activity involved in the glyoxylate shunt and methylisocitrate lyase activity responsible for catalyzing the last reaction of the MCC, resulting in the production of succinate and pyruvate that are routed into the TCA cycle (Lee et al., 2013). Although MCC is responsible for metabolizing the toxic intermediates produced from catabolism of odd-chain fatty acids (OCFAs) such as cholesterol, which is the primary source of nutrients during host infection, it has been observed that both the glyoxylate shunt and MCC are functional in wild-type Mtb, even in the presence of a glycolytic carbon source (Eoh and Rhee, 2014). Icl1-deficient Mtb undergoes a progressive depletion of TCA cycle intermediates, which in-turn impairs Mtb’s respiratory activity. Although excess of leucine in the extracellular environment is lethal, intracellular leucine production is critical as it provides an alternative pool for acetyl-CoA biosynthesis which feeds into the respiratory and lipid metabolic pathways (Ehebauer et al., 2015). We believe that the decline in intracellular leucine together with reduced expression of genes involved in its catabolism (*accD1*, *bkdA,* and *bkdB*) triggers the induction of alternate pathways such as MCC to maintain the cellular acetyl-CoA pool.

The complex cell-wall lipids of Mtb provide a hydrophobic barrier around the bacterium. These can be esterified with multiple MB long-chain fatty acids such as PDIM, sulfolipid-1, and di-, tri-, and poly-acyl trehalose. These MB lipids are synthesized by individual PKS complexes requiring malonyl-CoA and methyl malonyl-CoA (MMCoA), which are derived from acetyl-CoA and propionyl-CoA, respectively. PDIM is an important virulence factor and controls the movement of nutrients and large molecule inhibitors such as vancomycin across the bacterial cell membrane. While genes involved in the synthesis of the phthiocerol moiety of PDIM, such as *ppsB*, *ppsC,* and *ppsD,* are downregulated, those involved in the biosynthesis of SL-1 (*papA1* and *pks2*) and poly-acyl trehalose (*pks3*, *pks4*, *papA3*, and *fadD21*) are highly upregulated in drug-treated bacteria. Since both PDIM and SL-1 biosynthetic pathways share a common precursor, MMCoA, it has been observed that a reduction of PDIM synthesis favors SL-1 production due to increased flux of MMCoA to the SL-1 biosynthetic pathway, and vice versa (Jain et al., 2007). Intrigued by these observations, we speculate that semapimod induces remodeling of cell-wall lipids, restricting L-leucine uptake by an unknown mechanism (Figure 8). Targeting of PpsB by semapimod further supports the above hypothesis and warrants future studies to identify alterations in the cell envelope which impose restrictions on L-leucine transport.

Uptake of host-derived nutrients including amino acids supports bacterial viability during infection (Borah et al., 2019; Gouzy et al., 2014). Targeting the amino acid transport system offers a novel strategy to curtail the supply of host-derived nutrients to the pathogens, which are metabolically quiescent during intracellular stay (Cumming et al., 2018). Semapimod, which has been found to inhibit the L-leucine uptake in Mtb, may not be the preferred choice of L-leucine inhibitor owing to its anti-inflammatory effect. It inhibits the production of proinflammatory cytokines such as TNF-α, IL-1β, and IL-6. Also, it has been reported that semapimod interferes with the TLR4 signaling (Nishimatsu et al., 2008; Wehner et al., 2009; Wang et al., 2016; Miller et al., 2014). These cytokines play a critical role in providing protection to the host against active TB infection (Nosik et al., 2024). Nonetheless, despite its anti-inflammatory activity, which may promote bacterial proliferation, semapimod is able to reduce the intracellular burden of the virulent Mtb H37Rv strain in the lungs and spleen of the infected mice (Figure 7). These findings provide indirect evidence of the importance of L-leucine uptake for Mtb virulence during host infection.

Advancements in the genetic manipulation tools, such as CRISPR-Cas technology (Choudhary et al., 2015), have significantly contributed to enhancing our understanding of metabolic networks in mycobacteria. Screening of inhibitors against other Mtb auxotrophic strains is expected to provide novel insights into transport mechanisms of essential metabolites in Mtb that can be explored as novel anti-TB drug targets.

## Materials and methods

### Strains and culturing of bacteria

For propagation of plasmids, *Escherichia coli* strain XL1Blue (Agilent) was used, whereas expression and purification of PpsB was performed in *E. coli* BL21 DE3 (Novagen). Mtb H37Rv was received from Dr. Ramandeep Singh at THSTI, India, and Mtb mc^2^ 6206 strain was provided by Dr. William Jacobs at Albert Einstein College of Medicine, NY, USA. *E. coli* was cultured in the Luria-Bertani medium (Becton Dickinson), and Mtb H37Rv was cultured in Middlebrook 7H9 containing 0.05% tyloxapol (Merck), 0.5% glycerol (Merck), and 1 X OADS (oleic acid-albumin-dextrose-saline; 7H9-O) or Middlebrook 7H11 containing 0.5% glycerol (Merck) and 1 X OADS (7H11-O). For culturing Mtb mc^2^ 6206 strain, media were supplemented with 50 µg/mL L-leucine (Merck) and 24 µg/mL pantothenate (Merck) (7H9-PLO). Liquid cultures were grown either in Corning 50 mL centrifuge tubes (Corning) or in 250 mL flasks (Corning) with not more than one-third volume of bacterial cultures, whereas plates were incubated at 37 °C. Wherever required, we used 50 µg/mL kanamycin (Merck), 50 µg/mLl ampicillin (Merck), and 150 µg/mL hygromycin (Invitrogen) for *E. coli*, whereas for Mtb 25 µg/mL kanamycin and 50 µg/mL hygromycin were used.

### Screening of inhibitors

Repurposed library comprising of 3614 FDA-approved drugs was obtained from MedChemExpress (https://www.medchemexpress.com) in the 96-well plate format. All compounds were suspended in DMSO (Merck) at 5 mM concentration, and stored frozen at –80 C before use. For initial evaluation of compounds, 100 µL Mtb mc^2^ 6206 culture, diluted to OD_600_ of ~0.01 in 7H9-PLO medium was aliquoted in each well of the round-bottom 96-well plate containing 1 µL of respective drugs, so that final concentration of drug became 50 µM. Side wells were filled with 100 µL medium to maintain humidity and to prevent loss of samples from drug-containing wells. As an untreated (UT) control, 100 µL bacterial culture was treated with 1 µL DMSO. Plates were incubated at 37 °C for 2–3 weeks until UT wells exhibited substantial growth. Drugs showing complete inhibition of growth by visual inspection of the pellet formation at the bottom were considered inhibitors of Mtb mc^2^ 6206, and used for determination of drug concentration inhibiting 90% growth (MIC_90_).

### Determination of MIC_90_

The MIC_90_ of active inhibitors was determined by using the resazurin assay. Briefly, Mtb mc^2^ 6206 was incubated at OD_600_ of 0.005 with different concentrations of drug ranging from 25 to 0.10 µM in the flat bottom 96-well plates. After 2 weeks of incubation at 37 °C, cultures were transferred in the black 96-well plates and incubated with 10% of Alamar Blue reagent (Thermo Fisher Scientific) for 16 hr at 37 °C. Viability was calculated by comparing fluorescence of the drug-treated and DMSO-treated samples at 560 nm of excitation and 590 nm of emission. The minimum drug concentration at which ~90% growth inhibition is found, was considered as MIC_90_.

### Cell lines

The human monocytic leukemia cell line THP-1 (Sigma-Aldrich) was used in this study. The identity of the cells was authenticated by the provider via Short Tandem Repeat (STR) profiling using PCR. The observed STR loci profile was as follows: Amelogenin: X,Y; CSF1PO: 11,13; D13S317: 13; D16S539: 11,12; D5S818: 11,12; D7S820: 10; THO1: 8,9.3; TPOX: 8,11; and vWA: 16. Prior to experimentation, the cells were confirmed to be free of mycoplasma contamination.

### Macrophage infection and intracellular CFU analysis

For macrophage infection, THP1 cells were seeded in 24-well plates at a density of 2×10^5^ cells per well and differentiated using 20 nM phorbol 12-myristate 13-acetate (PMA). After 48 hr of differentiation, cells were washed once with incomplete RPMI medium (Himedia). Thereafter, cells were infected with Mtb mc^2^ 6206 at 1:5 MOI (macrophage: bacteria) using RPMI medium containing 10% FBS medium, 24 µg/mL pantothenate, and 50 µg/mL L-leucine (RPMI-PL). After 4 hr of incubation, cells were washed with 1 x PBS three times and replenished with RPMI-PL. After overnight incubation, cells were treated with 0.1, 0.2, and 1.0 µM semapimod, and 2.5 µM INH in four wells each, and the remaining four wells were left untreated. Drug treatment was repeated again after 3 days of incubation. On the 6th day, cells were lysed in 0.1% Triton X100, and serial dilutions of the lysates were plated on 7H11-OADS-PL agar plates for CFU enumeration.

### Global gene expression analysis by RNA sequencing

Freshly inoculated cultures of Mtb mc^2^ 6206 were treated with 50 nM of semapimod at 0.40 OD, whereas another set was left untreated. After 24 hr of incubation at 37 °C with shaking at 200 rpm, both semapimod-treated and untreated cultures were pelleted down, washed twice with 1 x PBS, and stored at –80 °C before RNA extraction. Total RNAs were extracted from three biological replicates of untreated (control) and semapimod-treated cultures followed by treatment with DNase I, as described previously (Pal et al., 2023). The DNase-treated RNA samples were supplied to Clevergene (https://clevergene.in/) for further processing and sequencing, typically as reported earlier (Pal et al., 2023). Genes that exhibit absolute log2 fold change ≥1, and FDR-adjusted p value of <0.01 were considered significant, and their expression profile is presented in the volcano plot and heatmap.

### Quantitative reverse transcription real-time PCR (qRT-PCR)

Total RNA was treated with DNase I (Ambion), and 500 ng of DNase-treated RNA samples were subjected to cDNA synthesis using SuperScript IV reverse transcriptase, as recommended by the manufacturer (Thermo Fisher Scientific). qRT-PCR was performed by using 50 ng cDNA and gene-specific primers (Supplementary file 1) with the help of SYBR Green PCR Master Mix (ABI). Amplification was monitored in real-time and quantification was carried out using ABI 7500 Fast Real-Time PCR System (Applied Biosystems), as mentioned earlier (Pal et al., 2023).

### Measurement of ATP

Mtb mc^2^ 6206 was cultured in the presence or the absence of 50 nM semapimod for 24 hr. For intracellular ATP estimation, 1 mL of untreated and treated cultures at OD_600_ of 0.1 were pelleted by centrifugation, washed twice with 1 x PBS, and lysed in 200 µL of 1 x PBS by boiling at 98 °C for 10 min. The bacterial lysates were centrifuged at 14,500 rpm for 3 min to remove debris, and supernatant was collected for ATP estimation by using Bac Titer-Glo assay kit as recommended by the manufacturer (Promega).

### Estimation of intracellular amino acids

Intracellular level of amino acids was estimated by liquid chromatography mass spectrometry (LC-MS/MS), as follows:

- *Preparation of samples:* For estimation in the presence of semapimod, freshly-grown Mtb mc^2^ 6206 cultures were diluted to 1.0 OD_600_ and divided into two portions: one treated with 50 ng/mL semapimod and the other one left untreated. After 24 hr of incubation at 37 °C with shaking at 200 rpm, cultures equivalent to 5.0 OD_600_ were drawn from both the samples and pelleted by centrifugation. To measure the levels of amino acids under regular conditions in the WT or the Sem^R^ strains of Mtb mc^2^ 6206, bacteria were freshly inoculated as 0.1 OD_600_ and cultured for 7 days at 37 °C with shaking at 200 rpm. Cultures equivalent to 5.0 OD_600_ were subsequently subjected to pelleting by centrifugation. After thorough washing with 1 x PBS, bacterial metabolites were extracted using the methanol precipitation method and subjected to LC-MS/MS.
- *LC-MS/MS:* QTRAP 6500+MS system coupled with ultra-high-performance liquid chromatographic system, (SCIEX ExionLC) was used for targeted LC-MS analysis. The ACQUITY UPLC HSS T3 column (1.8 µm, 2.1x100 mm; Waters) was used for separation, and the column oven temperature was set at 40 °C. Solvent A (10 mM ammonium formate with 0.1% formic acid in water) and solvent B (0.1% formic acid in methanol) were used for LC gradient with changing the concentration of solvent B as follows: 0 min, 1% B; 1 min, 15% B; 4 min, 35% B; 7 min, 95% B; 9 min, 95% B; 10 min, 1% B; 20 min, 1% B. The flow rate was kept at 300 µL/min and the injection volume was 5 μL. The following parameters were used for electrospray ionization: electrospray voltage, +5500 V; ion source temperature, 500 °C; curtain gas of 24, CAD gas 9, and gas 1 and 2 of 60 and 40 psi, respectively.
- *MRM parameters:* The following optimized MRM parameters of de-clustering potential (DP), collision energies (CE), entrance potential (EP), and collision cell exit potential (CXP) were used: DP-40, EP-10, CE-15, and CXP-22. Commercially procured ultrapure L-leucine, valine, and proline (Merck) were used for selection of Q1 and Q3 masses. Sharp peaks of pure compounds were chosen as references to identify the corresponding peaks in samples. Data analysis for LC-MS/MS was performed using the SCIEX (ANALYST 1.7.2) software. Analyte was confirmed by comparing the retention time and the ratio of characteristic transition between the test sample and the reference.

### Estimation of percent viability in response to vancomycin or semapimod treatment

Bacterial cultures were incubated at OD_600_ of 0.005 with different concentrations of vancomycin or semapimod in the flat bottom 96-well plates. After 2 weeks of incubation at 37 °C, growth was estimated by measuring OD_600_ of the cultures using a multi-mode plate reader, which was used for measuring percent viability in the drug-treated cultures with respect to the untreated control.

### Cloning and expression of genes in Mtb

The *leuC-leuD* and *panC-panD* ORFs were PCR amplified using Mtb H37Rv genomic DNA with gene-specific primer pairs (Supplementary file 1) and GoTaq polymerase, as recommended by the manufacturer (Promega). PCR amplicons with *Nde* I and *Hind* III overhangs were gel purified and subjected to restriction digestion with the respective enzymes. The digested DNA fragments were column-purified and annealed at the same sites in a Hyg^R^ replicative *E. coli*-mycobacterium shuttle plasmid, pHsp60 under a constitutive promoter of *hsp60* (Steyn et al., 2003)*.* The resulting recombinant plasmids, pHsp-*leuCD* and pHsp-*panCD* were electroporated in Mtb mc^2^ 6206 after sequence verification by Sanger’s sequencing. To ascertain expression of the respective genes, recombinant clones obtained by transformation with pHsp-*leuCD* were selected on 7H11-OADS agar plates containing pantothenate and hygromycin, whereas those obtained with pHsp-*panCD* were selected on 7H11-OADS agar plates containing L-leucine and hygromycin.

For expression of *ppsB*, the ~4.6 kb *ppsB* ORF was PCR amplified using Mtb H37Rv genomic DNA with Pr. 2396–2428 (PCR-A) and 2429–2397 (PCR-B; Supplementary file 1). Both the PCR-A and PCR-B fragments of ~2.2 and~2.4 kb, respectively, were subsequently cloned in the pGEMT-easy plasmid by following the TA cloning method, as suggested by the manufacturer (Promega). Both the recombinant clones were sequence verified, which revealed the presence of PCR-A in the forward orientation in pGEMT-A clone and PCR-B in the reverse orientation in pGEMT-B clone. Both the clones were subjected to restriction digestion with *Nde* I and *Sma* I. PCR-A fragment of ~2.2 kb was gel eluted and cloned in the pGEMT-B plasmid at the same sites, to create full-length ppsB of ~4.6 kb. Next, the DNA fragment comprising of full-length *ppsB* ORF was obtained from the positive clone of pGEMT-*ppsB* by *Nde* I*-Hind* III digestion and cloned in pHsp60 at the same sites. To express *mce1D* and *ppe60*, the respective ORFs were PCR amplified from Mtb H37Rv genomic DNA using Pr. 2277–2278 and Pr. 2281–2282, respectively, (Supplementary file 1), restriction digested, and cloned in pHsp60 at *Nde* I*-Hind* III sites, as described above. The resulting recombinant plasmids, pHsp-*ppsB*, pHsp-*mce1D,* and pHsp-*ppe60,* were verified by Sanger’s sequencing and electroporated in wild-type or Sem^R^ Mtb mc^2^ 6206. The transformants were selected on 7H11-PLO agar plates containing hygromycin, and positive clones verified by colony PCR were used in subsequent experiments.

### Generation of Sem^R^ strain

To induce semapimod-resistance, 5 mL culture of Mtb mc^2^ 6206 at OD_600_ of ~2.0 was pelleted by centrifugation followed by suspension in ~500 µL 7H9-PLO medium. Aliquots of 50 µL of the culture suspension were spread on multiple 7H11-PLO agar plates containing 100 nM semapimod. After 4–8 weeks of incubation at 37 °C, single colonies that appeared on drug-containing plates were cultured in 1 mL 7H9-PLO medium. Subsequently, cultures of the wild-type and putative Sem^R^ strain of Mtb mc2 6206 were spotted on 7H11-PLO agar plates with or without 50 nM semapimod to monitor growth.

### Genomic DNA extraction from mycobacteria and whole genome sequencing

Mtb mc^2^ 6206 wild type and the Sem^R^ strains were grown in 7H9-PLO, and genomic DNA extraction was performed using the conventional cetyltrimethylammonium bromide (CTAB)-based method. Whole genome sequencing (WGS) of the Mtb strains was conducted using the PacBio Sequel II long-read sequencing platform. The WGS was outsourced to Nucleome Informatics Private Limited, Hyderabad, India. A detailed sequencing workflow was employed to ensure the generation of high-quality data. Briefly, DNA quantification was performed using Qubit fluorometry, while DNA quality was assessed on a 1% agarose gel. Purity ratios were analyzed using the NanoDrop 2000 spectrophotometer. DNA fragments between 7 and 10 kb were prepared for SMRTbell library creation utilizing the Megaruptor 3 system, followed by purification with AMPure PB beads. The Agilent FEMTO Pulse analyzer was used to verify size distribution, and DNA concentration was measured with the DNA HS assay on the Qubit 3.0 fluorometer. Libraries were constructed with the Template Prep Kit, starting with the removal of single-strand overhangs, followed by DNA damage repair, and end-repair/A-tailing. Adapters were then ligated to the repaired DNA fragments to form SMRTbell libraries, with hairpin dimers removed using AMPure PB beads. The final pooled libraries were further purified and analyzed with the Agilent FEMTO Pulse. Primer annealing and polymerase binding were conducted using the Sequel II binding kit 2.2 before loading the libraries onto SMRT cells for sequencing in CCS/HiFi mode on the PacBio Sequel II system. This robust workflow ensured the production of high-quality sequencing data essential for precise genomic analysis.

### Bioinformatic analysis for genome assembly preparation and polymorphism detection

PacBio Sequel II raw subreads were processed into high-fidelity (HiFi) reads using the ‘CCS’ tool (version 6.2.0). The genome assembly was constructed using ‘Trycycler’ (version 0.5.4) with its default parameters (Wick et al., 2021). Three individual assemblies were initially created with the tools ‘Flye’ (version 2.9.2), ‘Minipolish’ (version 0.1.3), and ‘wtdbg2’ (version 2.5) (Khoshnood et al., 2021; Wick and Holt, 2019; Ruan and Li, 2020). These assemblies were combined to generate a consensus assembly using Trycycler’s default configuration. Genome completeness was evaluated using BUSCO (version 5.4.5), with the actinobacteria_class_odb10 lineage dataset as the reference (Simão et al., 2015). For comparative genomics, variant calling was performed using Genome Analysis Toolkit (GATK, version 4.5), FreeBayes (version 1.3.7), and TB-Profiler (version 5.0.1; Phelan et al., 2019; Garrison and Marth, 2012; McKenna et al., 2010). Mutations of interest were carefully selected based on their confidence levels to ensure accuracy.

### Cloning, expression, and purification of PpsB

Full-length *ppsB* ORF fragment was obtained from pGEMT-*ppsB* by *Nde I-Hind III* digestion and cloned at the same sites in pET28b (Novagen) to express the recombinant protein with 6 x His tags at the N-terminus *in E. coli*. For this, pET28b-*ppsB* was transformed in *E. coli* C43(DE3) cells and recombinant clones were selected on LB agar containing kanamycin. Single colony of *E. coli*::pET28b-*ppsB* was inoculated in 10 mL of LB broth medium containing kanamycin and culture was grown to turbidity at 37 °C with shaking at 200 rpm. Secondary culture was subsequently inoculated from the seed culture in 1 L of LB broth medium with kanamycin. Expression of 6XHis-PpsB was induced by adding 1 mM IPTG in the culture at OD_600_ of 0.80. After overnight incubation with IPTG at 18 °C, culture was pelleted and washed twice with 1 x PBS. Induced culture pellet was suspended in 50 mL of lysis buffer (50 mM Tris, pH 8.0, 50 mM NaCl, and 5% glycerol), and cell lysis was performed by using French press. Cell debris was removed by centrifugation and clarified cell lysate was incubated with Ni-NTA beads (Qiagen) for 3 hr at 4 °C. Beads were subsequently transferred into an empty column and unbound proteins were allowed to pass through. Beads immobilized with 6XHis-PpsB were washed with five bed volumes of the lysis buffer, followed by washing with five bed volumes of wash buffer (lysis buffer containing 20 mM imidazole). Immobilized 6XHis-PpsB was subsequently eluted with elution buffers (lysis buffer containing 100–300 mM imidazole). The purity of eluted protein in different elution fractions was assessed by SDS-PAGE. Fractions showing >90% purity of PpsB were pooled and dialyzed against lysis buffer. The purified proteins were stored in small aliquots at –80 °C for subsequent use.

### Protein-drug interaction by BLI-Octet

Optical interference-based Bio layer interferometry (BLI) from the Octet system by ForteBIO was used to ascertain the interaction of semapimod with Mtb PpsB. Briefly, purified 6xHis-tagged PpsB protein was dialyzed in 10 mM sodium acetate pH 3.9, followed by immobilization onto the amine reactive group sensor (second-generation; AR2G) up to a level of 2.1 nm. Different concentrations of semapimod were prepared in water and used to acquire a differential graded response. The binding constant was calculated as per the standard steps of baseline (60 s), association (180 s), and dissociation (180 s).

### Extraction of lipids

To obtain the apolar lipids, bacterial cultures equivalent to an OD_600_ of ~150 were pelleted, washed with 10 mL of water, and suspended in 2 mL methanol-0.3% NaCl (10:1) and 1 mL petroleum ether. After mixing vigorously to emulsify the organic and the aqueous layers, samples were centrifuged at a speed of 2000 × *g* for 5 min to allow the mixture to separate. The upper organic layer was collected in the microcentrifuge tubes, and the extraction was repeated by adding 1 mL petroleum ether to the lower aqueous phase and stirred for another 30 min. The organic layer was collected as described above and combined with the previous extracts. Petroleum ether was subsequently evaporated by vacuum drying at room temperature, and the resulting apolar lipids were stored at –80 °C for analysis by TLC.

To prepare MAMES, culture pellets equivalent to an OD_600_ of ~150 were suspended in 1 mL of 20% tetrabutylammonium hydroxide and incubated at 100 °C overnight. After overnight incubation, 0.4 mL of dichloromethane and 50 µL of iodomethane were added to the reaction mixture and stirred for 1 hr at room temperature. The biphasic mixture was separated by centrifugation at 2000 × *g* for 5 min, and the lower organic layer containing MAMES was collected in the microcentrifuge tubes. The MAMES fraction was completely dried and suspended in 1 mL of diethyl ether. The supernatant was collected in a new tube and vacuum dried. The dried fraction was suspended in 0.2 mL of toluene and 0.1 mL of acetonitrile, and after complete dissolution, 0.2 mL more acetonitrile was added. Samples were chilled at –20 °C overnight, and the MAMES were collected by centrifugation at 10,000 × *g* for 20 min. MAMES present in the pellet fraction were stored at –80 °C for further analysis by TLC.

### Analysis of lipids by TLC

Both the apolar lipids and MAMES were detected on aluminum-backed silica gel 60F254 plates (Merck). Dried lipid pellets were suspended in dichloromethane, and equal volumes of samples from both the strains were spotted on the TLC plates. Apolar lipids were resolved by two-dimensional TLC (2D TLC) using petroleum ether:ethyl acetate solvent (98:2) for three times in the first dimension, and petroleum ether:acetone mixture (98:2) for one time in the second dimension. MAMES were resolved by one-dimensional TLC using petroleum ether:diethyl ether (95:5) solvent for six times. After complete run, TLC plates were air dried and sprayed with 5% phosphomolybdic acid solution, prepared in ethanol. Lipids were visualized on the TLC plates after charring at 100 °C for 2–5 minutes.

### Mice infection

Mice infection was performed as per the guidelines by the committee for the purpose of control and supervision of experiments on animals (CPCSEA, India). Animal infection experiment was performed after receiving approval from the Institutional Animal Ethics Committee of THSTI (approval no. IAEC/THSTI/293). Every effort was made to minimize suffering during euthanasia.

Briefly, 6- to 8-week-old female BALB/c mice were infected with a single-cell suspension of mouse-passaged Mtb H_37_Rv at ~5 × 10^7^ cells/mL via aerosol route in a closed aerosol chamber in the biosafety level 3 laboratory. To check the establishment of infection, bacterial load in lungs was examined at day 1 post-infection, and the disease progression was assessed at day 21 post-infection, by CFU plating on selective 7H11-OADS agar plates containing 100 µg/mL carbenicillin, 22 µg/mL trimethoprim, 22 µg/mL amphotericin B, 11 µg/mL vancomycin, 27.5 µg/mL polymyxin B, and 0.88 µg/mL cycloheximide. At day 21, mice were divided into two groups: one group was given semapimod (5 mg/kg in 5% sucrose) by daily oral gavaging, whereas the other half was given an equal dose of 5% sucrose. CFUs were enumerated at day 28 post-semapimod or sucrose treatment to examine bacterial burden in lungs and in spleen for dissemination.

### Statistics and reproducibility

Some experiments were only performed with two biological replicates. Statistical analysis was performed with data obtained from 3 or more biological repeats by determining p values with the help of GraphPad Prism version 10.3.1 (464) software.

## Data Availability

Raw data of the RNASeq are available from the GEO database under project accession number GSE284673. Raw data of the whole genome sequence of Mtb mc2 6206 can be accessed from the Sequence Read Archive (SRA) database under the Bio-project PRJNA1118666. The following datasets were generated: AgarwalN 2025Effect of L-leucine uptake inhibitor on whole-genome transcriptomic profile of Mtb mc2 6206NCBI Gene Expression OmnibusGSE284673 AgarwalN 2025Whole genome sequencing of *Mycobacterium tuberculosis* H37Rv mc2-6206NCBI Sequence Read ArchivePRJNA1118666

## Additional files

Supplementary file 1. List of oligonucleotides. Shown are the oligonucleotides used in this study.

MDAR checklist
