# Supplementary material for: Identifying a novel mechanism of L-leucine uptake in Mycobacterium tuberculosis using a chemical genomic approach
Source: eLife. 2026 Sep 23;14:RP107025. doi: 10.7554/eLife.107025 (PMC13600695; doi:10.7554/eLife.107025)
Supplement: Supplementary file 3 [file elife-107025-supp1.docx]

| **Oligo ID** | **Sequence (5'–3')** | **Description** |
| --- | --- | --- |
| atpF_RTF | GGTGAAGTGAGCGCGATTGTCC | Forward oligo used in qRT-PCR-based expression analysis of Mtb atpF |
| atpF_RTR | ACCATAGCGTCACGTTCCCG | Reverse oligo used in qRT-PCR-based expression analysis of Mtb atpF |
| clpP1_RTF | AAGTGACTGACATGCGTTCG | Forward oligo used in qRT-PCR-based expression analysis of Mtb clpP1 |
| clpP1_RTR | AGAGGCTGATGTCCTTGCTG | Reverse oligo used in qRT-PCR-based expression analysis of Mtb clpP1 |
| FtsZ_RTF | CCGGGTCTAATCAACGTCGACTTCG | Forward oligo used in qRT-PCR-based expression analysis of Mtb ftsZ |
| FtsZ_RTR | TCGACATCAGCACGCCTTGC | Reverse oligo used in qRT-PCR-based expression analysis of Mtb ftsZ |
| icl_RTF | GCTTCTACCGCACCAAGA | Forward oligo used in qRT-PCR-based expression analysis of Mtb icl1 |
| icl_RTR | TCGAGGTGCTTTTTCCAGT | Reverse oligo used in qRT-PCR-based expression analysis of Mtb icl1 |
| kasA_RTF | AAGTGGGATCTAGCGGTCAAGA | Forward oligo used in qRT-PCR-based expression analysis of Mtb kasA |
| kasA_RTR | ATCCTCTCGGCTCCACCTAGAC | Reverse oligo used in qRT-PCR-based expression analysis of Mtb kasA |
| qcrC_RTF | GTGTTGCTGCTGATAGCGCTG | Forward oligo used in qRT-PCR-based expression analysis of Mtb qcrC |
| qcrC_RTR | GATCAGACTCGGCCCGTGGTC | Reverse oligo used in qRT-PCR-based expression analysis of Mtb qcrC |
| whiB7_RTF | TGTCGGTACTGACAGTCCCC | Forward oligo used in qRT-PCR-based expression analysis of Mtb whiB7 |
| whiB7_RTR | TATCTCACCACCCCAAACGC | Reverse oligo used in qRT-PCR-based expression analysis of Mtb whiB7 |
| ppsB_RTF | CGGTTGCGGTGGTTGGCATC | Forward oligo used in qRT-PCR-based expression analysis of Mtb ppsB |
| ppsB_RTR | CCCGCGGTGTGATACCGAAG | Reverse oligo used in qRT-PCR-based expression analysis of Mtb ppsB |
| mce1D_RTF | CTGACGAACAACACGGTGGTCG | Forward oligo used in qRT-PCR-based expression analysis of Mtb mce1D |
| mce1D_RTR | CAACTGAATGTTCCGCGACGC | Reverse oligo used in qRT-PCR-based expression analysis of Mtb mce1D |
| rrs_RTF | TCCGGCCACACTGGGACTGAGATAC | Forward oligo used in qRT-PCR-based expression analysis of Mtb rrs |
| rrs_RTR | TATTACCGCGGCTGCTGGCAC | Reverse oligo used in qRT-PCR-based expression analysis of Mtb rrs |
| sigA_RTF | CCATCCCGAAAAGGAAGACC | Forward oligo used in qRT-PCR-based expression analysis of Mtb sigA |
| sigA_RTR | TCGAGGTCTGGTTCAGCGTC | Reverse oligo used in qRT-PCR-based expression analysis of Mtb sigA |
| Pr. 2396 | NNCATATGATGCGAACGGCTTTCAGCCGG | For PCR amplification of ppsB ORF |
| Pr. 2428 | ACCCGGGCGATCAACTCATCG |  |
| Pr. 2429 | GCCGACCGAACAAATCGATG |  |
| Pr. 2397 | NNAAGCTTCATTGTGTTCCTCTTAGTCGTTC |  |
| leuC_ORF_F | NNCATATGGCCTTGCAGACCGGCGAG | For PCR amplification of leuC-leuD ORFs |
| leuD_ORF_R | NNAAGCTTTCAGGGGGCGGGTAGAGTGC |  |
| panC_ORF_F | NNCATATGACGATTCCTGCGTTCCATC | For PCR amplification of panC-panD ORFs |
| panD_ORF_R | NNAAGCTTCTATCCCACACCGAGCCGGGGGTC |  |
| Pr. 2277 | NNCATATGAGCACCATCTTTGATATCCGCA | For PCR amplification of mce1D ORF |
| Pr. 2278 | NNAAGCTTCATTGACCCCCTCCTGCCTCA |  |
| Pr. 2281 | NNCATATGGTGGATTTCGGGGCGTTACC | For PCR amplification of ppe60 ORF |
| Pr. 2282 | NNAAGCTTCTATCCGGCGGCCGGTGTGC |  |
