# Supplementary material for: Identifying a novel mechanism of L-leucine uptake in Mycobacterium tuberculosis using a chemical genomic approach
Source: eLife. 2026 Sep 23;14:RP107025. doi: 10.7554/eLife.107025 (PMC13600695; doi:10.7554/eLife.107025)
Supplement: Supplementary file 4 [file elife-107025-fig4-data1.docx]

|  | **Rifampicin** | **INH** |
| --- | --- | --- |
| **WT** | 0.11µM | 0.40µM |
| **Sem^R^** | 0.11µM | 0.40µM |
