# Supplementary material for: Identifying a novel mechanism of L-leucine uptake in Mycobacterium tuberculosis using a chemical genomic approach
Source: eLife. 2026 Sep 23;14:RP107025. doi: 10.7554/eLife.107025 (PMC13600695; doi:10.7554/eLife.107025)
Supplement: Supplementary file 5 [file elife-107025-fig6-data1.zip › Figure 6ΓÇô source data 1.pdf]

Sem^R

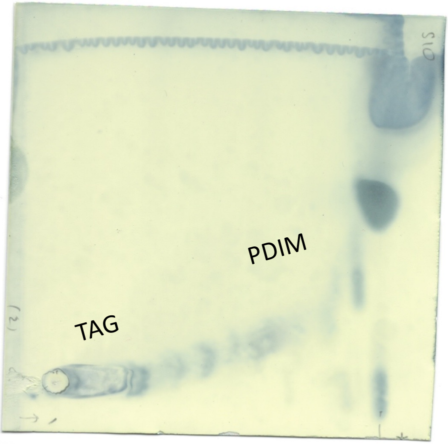

WT

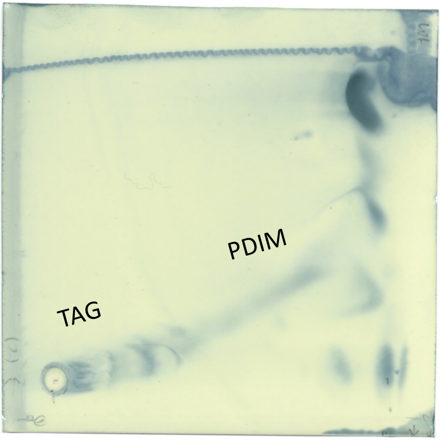

Figure 6–source data 1. Original TLC images corresponding to Figure 6, panel B. Positions of TAG and PDIM in Sem^R and WT strains are marked in the respective TLC images.
