# Supplementary material for: Identifying a novel mechanism of L-leucine uptake in Mycobacterium tuberculosis using a chemical genomic approach
Source: eLife. 2026 Sep 23;14:RP107025. doi: 10.7554/eLife.107025 (PMC13600695; doi:10.7554/eLife.107025)
Supplement: Supplementary file 7 [file elife-107025-fig6-figsupp1-data1.zip › Figure 6ΓÇöfigure supplement 1ΓÇösource data 1.pdf]

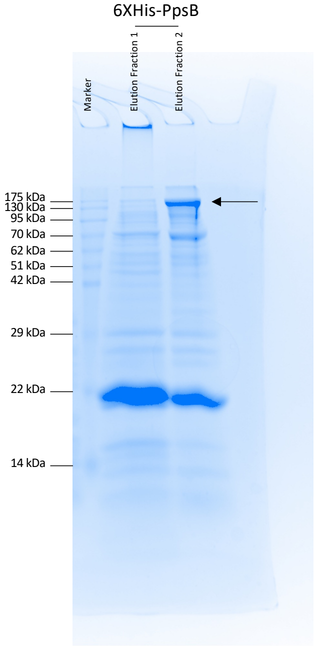

Figure 6—figure supplement 1—source data 1. Original Coomassie-stained polyacrylamide gel corresponding to Figure 6—figure supplement 1. Shown are two elution fractions. Pink plus molecular weight markers (Genedirex) were employed to ascertain the size of the purified protein. Arrow indicates the position of the 6XHis-PpsB.
