# Supplementary material for: Identifying a novel mechanism of L-leucine uptake in Mycobacterium tuberculosis using a chemical genomic approach
Source: eLife. 2026 Sep 23;14:RP107025. doi: 10.7554/eLife.107025 (PMC13600695; doi:10.7554/eLife.107025)
Supplement: Supplementary file 9 [file elife-107025-fig6-figsupp2-data1.zip › Figure 6ΓÇöfigure supplement 2ΓÇösource data 1.pdf]

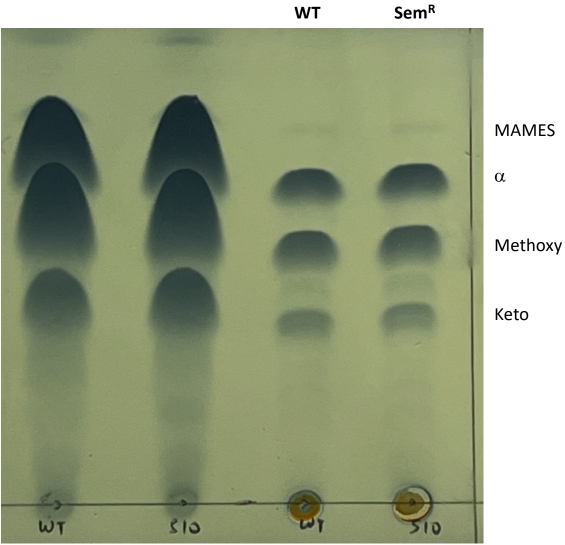

Figure 6—figure supplement 2—source data 1. Original TLC image corresponding to Figure 6—figure supplement 2. Positions of different MAMES in WT and Sem^R strains are marked.
