# Supplementary material for: Identifying a novel mechanism of L-leucine uptake in Mycobacterium tuberculosis using a chemical genomic approach
Source: eLife. 2026 Sep 23;14:RP107025. doi: 10.7554/eLife.107025 (PMC13600695; doi:10.7554/eLife.107025)
Supplement: Peer review history

# Editor report: eLife Assessment

**DOI:** 10.7554/eLife.107025.3.sa0

**Authors:** Amit Singh

**Affiliations:** Indian Institute of Science India

**Keywords:** Incomplete, Useful

By screening an FDA-approved small-molecule library against a leucine-dependent *M. tuberculosis* strain, this study identifies semapimod as an inhibitor of Mtb growth that functions by impairing leucine import. The work is **useful** in linking leucine uptake to cell wall lipid biology in Mtb. However, the mechanistic understanding remains **incomplete**. Additional experimental evidence is required to clarify how PDIM contributes to or regulates leucine uptake.

# Referee report: Reviewer #3 (Public review)

**DOI:** 10.7554/eLife.107025.3.sa1

Agarwal et al identified the small molecule semapimod from a chemical screen of repurposed drugs with specific antimycobacterial activity against a leucine-dependent strain of *M. tuberculosis*. To better understand the mechanism of action of this repurposed anti-inflammatory drug, the authors used RNA-seq to reveal a leucine-deficient transcriptomic signature from semapimod challenge. The authors then measured a decreased intracellular concentration of leucine after semapimod challenge, suggesting that semapimod disrupts leucine uptake as the primary mechanism of action. Unexpectedly however, resistant mutants raised against semapimod had a mutation in the polyketide synthase gene ppsB that resulted in loss of PDIM synthesis. The authors believe growth inhibition is a consequence of decreased accumulation of leucine as a result of an impaired cell wall and a disrupted, unknown leucine transporter. This study highlights the importance of branched-chain amino acids for *M. tuberculosis* survival and the chemical genetic interactions between semapimod and ppsB indicate that ppsB is a conditionally essential gene in a medium deplete of leucine.

The conclusions regarding the leucine and PDIM phenotypes are moderately supported by experimental data. The authors do not provide experimental evidence to support a specific link between leucine uptake and impaired PDIM production. Additional work is needed to support these claims and strengthen this mechanism of action.

A mechanistic gap still exists for the model of semapimod antitubercular activity. The basis for semapimod activity is that the leucine auxotroph strain cannot acquire leucine from its environment, and thus the bug ceases to grow. Under normal growth conditions, the leucine auxotroph strain produces PDIM and acquires exogenous leucine through some mechanism (either through a transporter or through PDIM). Semapimod binding to PpsB causes the cell to alter its PDIM profile (lacking experimental for this), and now with the altered PDIM profile the cell cannot acquire enough exogenous leucine to sustain growth (either because the altered PDIM profile interferes with the leucine transporter activity or through PDIM uptake). Acquiring a mutation in ppsB results in cells unable to produce PDIM (some evidence supporting this) but can now acquire enough exogenous leucine to sustain growth. I cannot find the connection between cells that have normal PDIM with normal leucine uptake and cells that are missing PDIM with normal leucine uptake.

(1) The manuscript would benefit from adding additional antibiotic controls to experiments. With the current experimental approaches, it is unclear if these signatures are the result of semapimod specifically or the effect of an antimicrobial agent. Adding additional strains to the 2D TLC experiments could provide more confidence in the absence or modifications of the PDIM band.

(2) The intriguing observation that wild-type H37Rv is resistant to semapimod but the leucine-auxotroph is sensitive should be further explored. If the authors are correct and semapimod does inhibit leucine uptake through a specific transporter or modified PDIM profiles, testing semapimod activity against the leucine-auxotroph in various concentrations of BCAAs could highlight the importance of intracellular leucine. Cells might recover growth in the presence of semapimod treatment if enough leucine is provided in the media and some fraction is able to enter the cell through the impaired PDIM barrier.

# Referee report: Reviewer #4 (Public review)

**DOI:** 10.7554/eLife.107025.3.sa2

Summary:

In this study, the authors screened an FDA-approved repurposed library of small-molecule inhibitors against the auxotrophic strain Mtb mc2 6206 and found that semapimod exclusively inhibited its growth. Further studies showed that it inhibits L-leucine uptake by interacting with PpsB, although the exact mechanism remains unknown. Interestingly, semapimod showed antibacterial activity against H37Rv only in vivo, not in vitro, suggesting a dependence on host-derived exogenous leucine during intracellular growth. This work therefore suggests that uptake of host-derived leucine can be targeted as an effective strategy to reduce intracellular survival of Mtb.

Strengths:

The authors have used different approaches to understand the mechanism of L-leucine uptake in Mtb. To start, they conducted an in vitro screen using an FDA-approved library, followed by transcriptomic and metabolic analyses of different Mtb mutants. Through whole-genome sequencing, they identified mutations conferring resistance to semapimod to gain further mechanistic understanding. This led to the analysis of semapimod-PpsB interaction by BLI-Octet and analysis of cell-wall apolar lipid, which explained how PDIM loss resulted in sensitivity to vancomycin. Finally, infection experiments in mice surprisingly showed that semapimod was effective against intracellular Mtb in vivo but not in vitro.

Weakness:

The major weakness of this study is that it is unclear what role PpsB plays in L-leucine uptake. It is also not clear why intracellular Mtb relies on exogenous leucine rather than endogenous leucine. Does intracellular Mtb lose its ability to synthesize leucine, which is why semapimod is active in vivo but not in vitro? Or semapimod has any other effect on host immunity that has not been explored. I have a few minor comments, which are as follows:

(1) Authors state that "The colony forming unit (CFU) estimation further shows a bactericidal activity of this molecule which causes 88% reduction of bacterial viability on day 2 and >99% reduction after 5 days of incubation" (Fig. 1d). However, this is only true when compared to the untreated control. Compared to the Day 0 control, treated bacteria appear to have undergone little or no change, suggesting that the compound is bacteriostatic, not bactericidal. The drug concentration used for Fig 1d is not mentioned. For Fig. 1e, there is no day 0 control, and the comparison is with the untreated control at Day 6, which again does not suggest bactericidal action of Semapimod.

(2) The authors report that "Notably, no cytotoxic effect was observed at this concentration against THP1, thus ruling out the possibility of cell lysis by semapimod," but the data are not shown. Similarly, authors state that "As a control, interaction of semapimod was also analyzed with the purified Ppe60, which fails to exhibit any binding," but the data is not shown.

(3) Line 235: change "promote" to "promoter".

# Referee report: Reviewer #5 (Public review)

**DOI:** 10.7554/eLife.107025.3.sa3

Summary:

The authors have extensively characterized the response of the leucine and pantothenate auxotroph Mtb strain H37Rv mc26 206 to an FDA-approved compound library and identified semapimod that is, at best, bacteriostatic in its action against the pathogen. The authors have used transcriptional profiling, metabolite quantification and a screening of genetically-resistant mutants to identify changes in leucine uptake under semapimod exposure. Based on these data, the authors attribute changes in antibiotic susceptibility to differences in environmental leucine availability and bacterial PDIM architecture. While the work presents an interesting avenue of investigation of metabolite uptake and utilization in a comparative fashion between fully virulent and auxotroph Mtb strains, it lacks clear and direct evidence to link the observations with a mechanistic explanation.

Strengths:

The authors used a well-designed screening strategy for FDA-approved compounds against a metabolically defined strain and follow up characterization of semapimod exposure through RNA-seq and pathway analysis, metabolomics and time-course analysis of drug effects. The data has been interestingly interpreted to identify a phenotypic connection between PDIM and altered drug susceptibility.

Weaknesses:

The major gap in the study is the speculative nature of the mechanism underpinning the connection between PDIM architecture and changes in leucine uptake under various bacterial growth conditions.

(1) Despite claims of identifying a "novel leucine uptake mechanism", the authors only provide endpoint metabolite measurements rather than kinetic leucine transport studies.

(2) A clear explanation for the differences in susceptibility between auxotroph and fully virulent Mtb strains through changes in "PDIM architecture" is not supported by any direct evidence such as structural analysis, lipidomics, or direct measurement of PDIM architectural changes.

(3) The figures 1D (lines 110-112, "kills bacteria") and 7c (lines 283-285) are used to infer a bactericidal role of semapimod, which maybe a mischaracterization of drug activity. The trend in CFUs in both cases seems of no bacterial growth rather than a CFU reduction- therefore interpreted as "bacteriostatic" at best. These observations would in fact align with the general antibiotic/stress response signature identified by RNA-seq, where leucine transport related genes only happen to be a small subset of many dysregulated genes. How do the authors disentangle these generic signatures from the leucine transport evidence, other than endpoint metabolite quantification?

(4) Furthermore, the studies with supplementation of leuCD (and not panCD) in rescuing from semapimod susceptibility are not supported by a clear mechanistic link. The complementation of leuCD does not completely rescue growth- does this indicate differences in uptake and metabolism? The authors should test this by monitroing the growth of the strains in minimal medium in presence and absence of exogenous leucine.

(5) It remains unclear if the authors attribute leucine uptake differences to a loss of PDIM or changes in PDIM amount and architecture. No direct evidence is provided for differences in PDIM production in the WT H37Rv strain and the auxotroph mc2 6206 strains used in this study. Mulholland et al (2024) report similar PDIM levels for WT and auxotrophic Mtb (mc2 6206) in their stocks passaged to maintain PDIM. This could change for stocks maintained differently. Since the presence of PDIM has classically been used to explain a penetration barrier for small molecules and the schematic provided by the authors at the end of the manuscript (figure 8c) suggest free leucine penetration in the absence of PDIM, how do the authors explain the increased leucine uptake and sensitivity of a PDIM positive auxotroph to semapimod through direct experimental evidence? Further on the point of PDIM production, the WT auxotroph strain seems to produce limited amounts of PDIM as evidenced by the TLC data in Figure 6b. To solidify this point, the authors should test other point mutants for PDIM production (not attenuated for growth) through TLC and quantify these differences. These data should be compared with PDIM production in the WT Mtb H37Rv strain (used by the authors) under in vitro growth conditions. A comparative lipidomics of cell envelope components might be insightful in explaining these differences. I believe answering this query is crucial and within the scope of the work whose central claim is the identification of a novel leucine uptake mechanism. It would be interesting, in fact, to identify a novel transporter associated with the PDIM layer on the cell envelope.

# Author comment: Author response

**DOI:** 10.7554/eLife.107025.3.sa4

**Authors:** Nisheeth Agarwal, Himanshu Gogoi, Eeba Eeba, Linus Augustin, Mohd Younus Khan, Yashwant Kumar, Sayan Kumar Bhowmick, Bappaditya Dey

**Affiliations:** Translational Health Science and Technology Institute Faridabad India; Translational Health Science and Technology Institute Faridabad India; Translational Health Science and Technology Institute Faridabad India; Translational Health Science and Technology Institute Faridabad India; Translational Health Science and Technology Institute Faridabad India; Translational Health Science and Technology Institute Faridabad India; National Institute of Animal Biotechnology Hyderabad India; National Institute of Animal Biotechnology Hyderabad India

The following is the authors’ response to the original reviews.

**Public Reviews:**

**Reviewer #1 (Public review):**

Summary:

In this manuscript, the authors used a leucine/pantothenate auxotrophic strain of Mtb to screen a library of FDA-approved compounds for their antimycobacterial activity and found significant antibacterial activity of the inhibitor semapimod. In addition to alterations in pathways, including amino acid and lipid metabolism and transcriptional machinery, the authors demonstrate that semapimod treatment targets leucine uptake in Mtb. The work presents an interesting connection between nutrient uptake and cell wall composition in mycobacteria.

Strengths:

(1a) The link between the leucine uptake pathway and PDIM is interesting but has not been characterized mechanistically. The authors discuss that PDIM presents a barrier to the uptake of nutrients and shows binding of the drug with PpsB. However it is unclear why only the leucine uptake pathway was affected.

We observe interference of L-leucine, but not of pantothenate, uptake in mc2 6206 strain upon semapimod treatment. At present, we do not have any clue whether PDIM presents a barrier exclusively to the uptake of L-leucine. Further studies may shed a light on underlying mechanism(s) by which L-leucine uptake is modulated by this small molecule.

(1b) We still do not know what PpsB actually does for amino acid uptake - is it a transporter?

By BLI-Octet we do not find any interaction between L-leucine and PpsB. Therefore, we doubt that PpsB is a transporter of L-leucine.

(1c) Does semapimod binding affect its activity?

Our study suggests that semapimod treatment alters PDIM architecture which becomes restrictive to L-leucine. However, at present the exact mechanism is not clear. Further studies are required to thoroughly examine the effect of semapimod on Mtb PpsB activity and alterations in PDIM by mass spectrometry.

(1d) Does the auxotrophic Mtb have lower PDIM levels compared to wild-type Mtb?

As per the published report by Mulholland et al, and by vancomycin susceptibility phenotype in our study, both the strains appear to have comparable PDIM levels.

(2) The authors show an interesting result where they observed antibacterial activity of semapimod against H37Rv only in vivo and not in vitro. Why do the authors think this is the basis of this observation? It is possible semapimod has an immunomodulatory effect on the host since leucine is an essential amino acid in mice. The authors could check pro-inflammatory cytokine levels in infected mouse lungs with and without drug treatment.

Semapimod inhibits production of proinflammatory cytokines such as TNF-α, IL-1β, and IL-6, which would indeed help pathogen establish chronic infection. However, a significant reduction in bacterial loads in lungs and spleen upon semapimod treatment despite inhibition of proinflammatory cytokines clearly indicates bacterial dependence on host-derived exogenous leucine during intracellular growth.

(3) The authors show that the semapimod-resistant auxotroph lacks PDIM. The conclusions would be further strengthened by including validations using PDIM mutants, including del-ppsB Mtb and other genes of the PDIM locus, whether in vivo this mutant would be more susceptible (or resistant) to semapimod treatment.

PDIM is a virulence factor, and plays an important role in the intracellular survival of the TB pathogen. Mtb strains lacking PDIM are expected to show attenuated growth during infection, even without semapimod treatment. In such a case, it might be difficult to draw any conclusions about the effect of semapimod against PDIM(-) strains in vivo.

(4) Prolonged subculturing can introduce mutations in PDIM, which can be overcome by supplementing with propionate (Mullholland et al, Nat Microbiol, 2024). Did the authors also supplement their cultures with propionate? It would be interesting to see what mutations would result in Semr strains with propionate supplementation along with prolonged semapimod treatment.

Considering the fact that extensive subculturing may result in loss of PDIM, we avoided prolonged subculturing of bacteria. As presented in Fig. 6b, the WT bacteria retain PDIM. While performing the initial screening of drugs, we did not anticipate such phenotype, and hence bacteria were cultured in regular 7H9-OADS medium without propionate supplementation.

A comprehensive future study would help examining the effect of propionate on generation of semapimod resistant mutants in Mtb mc2 6206.

Weaknesses:

I have summarized the limitations above in my comments. Overall, it would be helpful to provide more mechanistic details to study the connection between leucine uptake and PDIM.

**Reviewer #2 (Public review):**

Summary

This important study uncovers a novel mechanism for L-leucine uptake by *M. tuberculosis* and shows that targeting this pathway with 'Semapimod' interferes with bacterial metabolism and virulence. These results identify the leucine uptake pathway as a potential target to design new anti-tubercular therapy.

Strengths

The authors took numerous approaches to prove that L-leucine uptake of *M. tuberculosis* is an important physiological phenomenon and may be effectively targeted by 'Semapimod'. This study utilizes a series of experiments using a broad set of tools to justify how the leucine uptake pathway of *M. tuberculosis* may be targeted to design new anti-tubercular therapy.

Weaknesses

(1) The study does not explain how L-leucine is taken up by *M. tuberculosis*, leaving the mechanism unclear. Even though 'Semapimod' binds to the PpsB protein, the relevant connection between changes in PDIM and amino acid transport remains incomplete.

While Leucine uptake involves specific transporters in other bacteria, such transport system is not known in Mtb. By screening small molecule inhibitors, we came across a molecule, semapimod, which selectively kills the leucine auxotroph (mc2 6206), but not the WT Mtb. To understand the underlying mechanism of differential susceptibility of the WT and auxotrophic strains to this molecule, we evaluated the effect of restoration of *leuCD* and *panCD* expression on susceptibility of the auxotrophic strain to semapimod. Interestingly, our results demonstrated that upon endogenous expression of *leuCD* genes, mc2 6206 strain becomes resistant to killing by semapimod. In contrast, no effect of *panCD* expression was observed on semapimod susceptibility of mc2 6206. These findings were further substantiated by gene expression analysis of semapimod treated mc2 6206, which exhibits differential regulation of a set of genes that are altered upon leucine depletion in Mtb as well as in other bacteria. Overall results thus provide first evidence of perturbation of L-leucine uptake by semapimod treatment of the leucine auxotroph.

To further gain mechanistic insights into the effect of semapimod on leucine uptake in Mtb, we generated the semapimod resistant strain which exhibits point mutation in 4 genes including *ppsB*. Interestingly, overexpression of wild-type *ppsB*, but not of other genes, restored susceptibility of the resistant bacteria to semapimod. Our observations that semapimod interacts with PpsB, and semapimod resistant strain accumulates mutation in PpsB resulting in loss of PDIM together support the involvement of cell-wall PDIM in regulation of L-leucine transport in Mtb.

As mentioned above, we anticipate that semapimod treatment brings about certain modifications in PDIM which becomes more restrictive to L-leucine. A comprehensive future study will be helpful to examine the effect of semapimod on Mtb physiology.

(2) Also, the fact that the drug does not function on WT bacteria makes it a weak candidate to consider its usefulness for a therapeutic option.

We agree that semapimod is not an appropriate drug candidate against TB owing to its inhibitory effect on production of proinflammatory cytokines such as TNF-α, IL-1β, and IL-6 that help pathogen establish chronic infection. However, a significant reduction in bacterial loads in lungs and spleen upon semapimod treatment despite inhibition of proinflammatory cytokines clearly indicates bacterial dependence on host-derived exogenous leucine during intracellular growth. Therefore targeting L-leucine uptake can be a novel therapeutic strategy against TB.

**Reviewer #3 (Public review):**

(1) Agarwal et al identified the small molecule semapimod from a chemical screen of repurposed drugs with specific antimycobacterial activity against a leucine-dependent strain of *M. tuberculosis*. To better understand the mechanism of action of this repurposed anti-inflammatory drug, the authors used RNA-seq to reveal a leucine-deficient transcriptomic signature from semapimod challenge. The authors then measured a decreased intracellular concentration of leucine after semapimod challenge, suggesting that semapimod disrupts leucine uptake as the primary mechanism of action. Unexpectedly, however, resistant mutants raised against semapimod had a mutation in the polyketide synthase gene ppsB that resulted in loss of PDIM synthesis. The authors believe growth inhibition is a consequence of decreased accumulation of leucine as a result of an impaired cell wall and a disrupted, unknown leucine transporter. This study highlights the importance of branched-chain amino acids for *M. tuberculosis* survival, and the chemical genetic interactions between semapimod and ppsB indicate that ppsB is a conditionally essential gene in a medium depleted of leucine.

The conclusions regarding the leucine and PDIM phenotypes are moderately supported by experimental data. The authors do not provide experimental evidence to support a specific link between leucine uptake and impaired PDIM production. Additional work is needed to support these claims and strengthen this mechanism of action.

As mentioned above, overall results from this study provide first evidence of perturbation of L-leucine uptake by semapimod treatment of the leucine auxotroph. Our observations that semapimod interacts with PpsB, and semapimod resistant strain accumulates mutation in PpsB resulting in loss of PDIM together support the involvement of cell-wall PDIM in regulation of L-leucine transport in Mtb.

As hitherto mentioned, it appears that semapimod treatment brings about certain modifications in PDIM which becomes restrictive to L-leucine. Future studies are required to gain detailed mechanistic insights into the effect of semapimod on Mtb physiology.

(2) Since leucine uptake and PDIM synthesis are important concepts of the manuscript, experiments would benefit from exploring other BCAAs to know if the phenotypes observed are specific to leucine, and adding additional strains to the 2D TLC experiments to provide confidence in the absence of the PDIM band.

We thank the peer reviewer for this suggestion. We would be happy to analyse the effect of semapimod on the level of other amino acids including BCAA by mass spectrometry.

(3) The intriguing observation that wild-type H37Rv is resistant to semapimod but the leucine-auxotroph is sensitive should be further explored. If the authors are correct and semapimod does inhibit leucine uptake through a specific transporter or disrupted cell wall (PDIM synthesis), testing semapimod activity against the leucine-auxotroph in various concentrations of BCAAs could highlight the importance of intracellular leucine. H37Rv is still able to synthesize endogenous leucine and is able to circumvent the effect of semapimod.

We thank the peer reviewer for this suggestion. We would explore the possibility of analysing the effect of increasing concentrations of BCAAs on mc2 6206 susceptibility to semapimod.

**Recommendations for the authors:**

(1A) Intracellular leucine can decrease from:

inhibition of transport/uptake via semapimod as the authors claim or

decreased uptake/requirement of many metabolites due to cells entering static growth arrest from challenge by semapimod

To rule out the growth-inhibitory effect of semapimod on L-leucine uptake, we estimated intracellular L-leucine in Mtb after brief exposure of 24 hours to 50ng/ml semapimod (kindly refer Materials and Methods). We confirmed that 24 hours of treatment with 50ng/ml semapimod does not cause cells entering static growth arrest.

(1B) increased consumption/utilization of leucine for some programmed response to semapimod challenge

Our results show reduced expression of genes involved in leucine catabolism such as accD1, bkdA and bkdB in semapimod-treated cells, and thus the above hypothesis seems unlikely.

(1C) Additional metabolites should be measured to determine the specificity of the semapimod challenge.

As mentioned below, we measured intracellular valine in the semapimod-treated Mtb 6206 by LC-MS/MS, which shows no change in its level. These observations thus corroborate a specific effect of semapimod on L-leucine level in the cell.

(2) The effect of Semapimod on L-leucine uptake is largely based on indirect evidence, without showing reduced transport of the amino acid. Gene expression data is not enough to prove that the amino acid transport is blocked. More compelling evidence is required to confirm this mechanism.

The authors could perform leucine uptake assays to directly confirm the functioning of Semapimod, inhibiting L-leucine transport. Another possibility would be to try out measuring intra-bacterial leucine levels for drug-treated versus untreated *M. tuberculosis* strains.

Data presented in the Fig. 3b shows lesser intracellular L-leucine upon semapimod treatment; in contrast, Sem^R^ strain exhibits ~3-fold more intracellular L-leucine, as estimated by mass spectrometry (kindly refer our response to comment #6 below). Together, these observations indicate an inhibitory effect of semapimod on L-leucine uptake by the auxotroph.

(3) The authors show that the overexpression of leuC-leuD restores Semapimod resistance in the auxotroph (Figs. 3C-3E). Is it possible to examine Semapimod resistance of WT-H37Rv or the complemented mutant grown in leucine-limiting conditions? This sort of evidence will be more direct on the specific drug-target beyond the auxotroph (mc^2^ 6206).

Because endogenous L-leucine synthesis pathway is functional in WT-H37Rv, as well as complemented auxotrophic strain, leucine-limiting conditions are unexpected to yield any effect on susceptibility to semapimod.

**Author response image 1.**

(4) Biolayer Interferometry (BLI) shows Semapimod binds to PpsB (Fig. 6); however, there is no clear evidence that it disrupts PDIM synthesis. More direct evidence would be to study the effect of Semapimod on a ppsB mutant (may be a knock-down). This would prove the specificity of Semapimod for PpsB. Likewise, it would be worth looking into the effect of Semapimod using mutant *M. tuberculosis* defective for PDIM synthesis.

As recommended by the peer reviewer, we created the *ppsB* knockdown strain in the Mtb mc2 6206 by CRISPRi and examined its vulnerability to semapimod treatment. As can be seen in the Author response image 1, *ppsB* KD strain shows lesser susceptibility to semapimod when compared with the pDcas9-control strain which exhibits significant growth inhibition on the 7H11-OADS-PL agar plate containing 200nM semapimod.

(5) Metabolomics experiments would benefit from including other control BCAAs like isoleucine and valine to determine if decreased intracellular levels of leucine are specific to semapimod or a general consequence of growth arrest from an antimicrobial agent.

As suggested by the reviewer, we measured intracellular valine as well as proline levels in the semapimod-treated Mtb 6206 by LC-MS/MS; data presented in the supplimentry figure 5 clearly show no change in their levels upon semapimod treatment.

(5) Figure 3c, pyrazinamide susceptibility assay could be included on the panCD strain to ensure complementation leads to functional panCD. Parent strain would be resistant to PZA, complement strain would be susceptible. (doi: 10.1038/s41467-019-14238-3).

The wild-type Mtb 6206 is unable to grow in the absence of pantothenate. We verified resumption of growth of Mtb 6206 in 7H9-OADS-L-leucine medium lacking pantothenate upon PanCD overexpression, which provides more direct evidence of the expression of functional copies of *panCD* genes.

(6) does the Sem-R mutant have increased levels of leucine?

As can be seen in the supplimentry figure 7, Sem^R^ strain shows ~3.0 fold increase in the intracellular L-leucine level when compared with the WT strain. In contrast, a comparable level of another BCAA– valine, is observed in both the strains
